# Single-cell alternative polyadenylation analysis delineates GABAergic neuron types

**DOI:** 10.1186/s12915-021-01076-3

**Published:** 2021-07-23

**Authors:** Yang Yang, Anirban Paul, Thao Nguyen Bach, Z. Josh Huang, Michael Q. Zhang

**Affiliations:** 1 Present Address: Department of Pharmacology, Tianjin Key Laboratory of Inflammation Biology, School of Basic Medical Sciences, Tianjin Medical University, Tianjin, 300070 China; 2 Department of Biological Sciences, Center for Systems Biology, The University of Texas at Dallas, Richardson, TX 75080 USA; 3 Cold Spring Harbor Laboratory, Harbor, Cold Spring, NY 11724 USA; 4 Deparment of Neural and Behavioral Sciences, Penn State College of Medicine, Hershey, PA 17033 USA; 5 Deparment of Neurobiology, Duke University Medical Center, Durham, NC USA

**Keywords:** Alternative polyadenylation, scRNA-seq, GABAergic neuron

## Abstract

**Background:**

Alternative polyadenylation (APA) is emerging as an important mechanism in the post-transcriptional regulation of gene expression across eukaryotic species. Recent studies have shown that APA plays key roles in biological processes, such as cell proliferation and differentiation. Single-cell RNA-seq technologies are widely used in gene expression heterogeneity studies; however, systematic studies of APA at the single-cell level are still lacking.

**Results:**

Here, we described a novel computational framework, SAPAS, that utilizes 3′-tag-based scRNA-seq data to identify novel poly(A) sites and quantify APA at the single-cell level. Applying SAPAS to the scRNA-seq data of phenotype characterized GABAergic interneurons, we identified cell type-specific APA events for different GABAergic neuron types. Genes with cell type-specific APA events are enriched for synaptic architecture and communications. In further, we observed a strong enrichment of heritability for several psychiatric disorders and brain traits in altered 3′ UTRs and coding sequences of cell type-specific APA events. Finally, by exploring the modalities of APA, we discovered that the bimodal APA pattern of *Pak3* could classify chandelier cells into different subpopulations that are from different laminar positions.

**Conclusions:**

We established a method to characterize APA at the single-cell level. When applied to a scRNA-seq dataset of GABAergic interneurons, the single-cell APA analysis not only identified cell type-specific APA events but also revealed that the modality of APA could classify cell subpopulations. Thus, SAPAS will expand our understanding of cellular heterogeneity.

**Supplementary Information:**

The online version contains supplementary material available at 10.1186/s12915-021-01076-3.

## Background

Alternative cleavage and polyadenylation of pre-mRNA is a process that generates diverse mRNA isoforms with different 3′-ends [1, 2]. APA is a pervasive post-transcriptional regulatory mechanism as approximately 70% of mammalian protein-coding genes contain multiple polyadenylation sites (poly(A) sites) [3, 4]. As post-transcriptional regulation events, APA contributes extensively to the diversity of the 3′ untranslated regions (3′ UTR) that harbor cis-regulatory elements interacting with RNA-binding proteins and/or microRNAs [5–8]. Through this mechanism, APA has been implicated in the regulation of mRNA degradation rates, translation efficiency, transport, and localization [7, 9–11].

Accumulated case studies of specific genes have validated the important roles of APA in numerous biological processes including cell differentiation, tumorigenesis, neuron activation, and cell reprogramming [11–17]. For example, previous studies reported that several oncogenes in cancer cells exhibit 3′ UTR shortening [13, 17]. The short 3′ UTR isoform of mRNA encoding insulin-like growth factor 2 mRNA binding protein 1 (*IGF2BP1*) shows increased mRNA stability and produces a higher abundance of proteins. Moreover, expressing the short isoform could promote oncogenic transformation, thereby linking APA with cancer development [17]. Another intriguing example is the *Bdnf* gene encoding the brain-derived neurotrophic factor that is subjected to APA and contains two different 3′ UTR isoforms with distinct functions in neurons. The short *Bdnf* isoform is restricted to the somata, whereas the long *Bdnf* isoform is localized in the dendrites. Mice lacking the long isoform exhibit deficits in pruning and enlargement in the dendritic spine and decreased synaptic plasticity in hippocampal neurons [18, 19].

To facilitate a deep understanding of APA at a genome-wide scale, several high-throughput sequencing techniques have been developed to capture the 3′-end of mRNAs, such as PAS-seq [20], 3′READS [4], 3′-seq [21], and PolyA-seq [3]. In addition, several bioinformatic methods have been developed to examine APA using conventional RNA-seq data, such as DaPars, APAtrap, and QAPA [13, 22, 23]. Recently, varieties of single-cell RNA-seq (scRNA-seq) techniques have emerged as powerful tools that allow us to characterize the transcriptional landscape at the resolution of individual cells. Moreover, the amount of scRNA-seq data from various tissues of different species increases at an unprecedented pace. Among these scRNA-seq protocols, 3′-tag-based scRNA-seq protocols provide us opportunities to analyze APA at the single-cell level as they are based on sequencing of the 3′-end of the RNA molecules.

In this study, we have developed a bioinformatics framework called SAPAS (Systematic Alternative Polyadenylation Analysis at Single-cell level) to characterize the alternative polyadenylation landscape by leveraging 3′-tag-based scRNA-seq data. SAPAS could be utilized to identify poly(A) sites, quantify APA events, and detect cell type-specific APA events. To demonstrate the effectiveness of our method, multiple lines of evidence were presented. In addition, we employed SAPAS to profile the APA landscape of six different GABAergic interneuron types in the mouse cerebral cortex. The results suggested that APA occurs in a cell type-specific manner. Remarkably, those identified genes with cell type-specific APA events are related to synaptic vesicle cycling, neurotransmitter release, ion transport, and cell respiration, suggesting that APA is involved in shaping synaptic communication and neuron identity determination. Furthermore, we found 3′ UTR of the neuron type-specific APA genes are significantly enriched for schizophrenia and intelligence heritability. Finally, we sought to explore the modality of APA in GABAergic neurons. Among the cortical interneurons, chandelier cells (CHCs) are a unique type of GABAergic interneuron with specific spatial and temporal origins, target the axon initial segment of pyramidal neurons and implicated in brain disorders, including schizophrenia, epilepsy, and autism spectrum disorder [24–26]. The results of modality analysis showed that the bimodal APA pattern of *Pak3* could demarcate subpopulations of CHCs that are from different laminar positions. This study provides insight into the understanding of APA regulation at the single-cell level and demonstrates a reliable computational method for APA analysis using scRNA-seq data.

## Results

### 3′-tag-based scRNA-seq data could be applied to poly(A) site identification

**Fig. 1:**
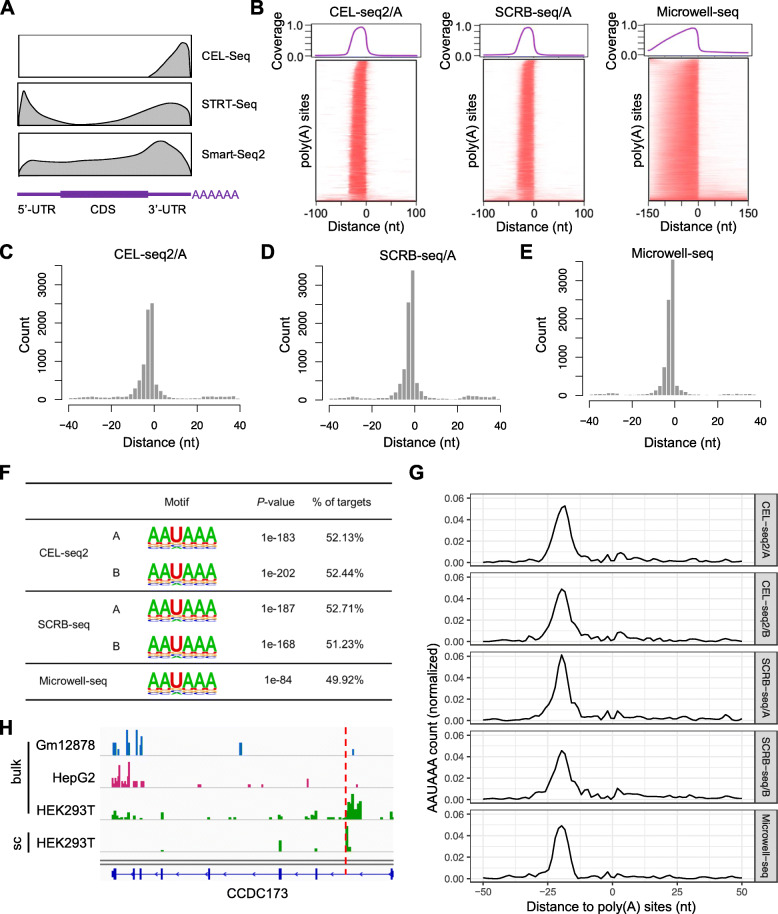
Identification of poly(A) sites using 3′-tag-based scRNA-seq data. **a** The schematic diagram depicts the read distribution along the gene model for different scRNA-seq methods, including the tag-based methods, including STRT-seq and CEL-seq, and the full transcript method, such as Smart-seq2. **b** The plots depict the read coverage of poly(A) reads around poly(A) sites annotated in GENCODE, including canonical and variants, in different scRNA-seq datasets, including CEL-Seq2/A dataset, SCRB-seq/A dataset, and Microwell-seq dataset. The upper panels depict the average read coverage of poly(A) reads around poly(A) sites. Y-axis: the average read coverage; X-axis: the distance from upstream 100 nt to downstream 100 nt to annotated poly(A) sites. The lower panels show the read coverage for each poly(A) site using heatmaps. Additional examples are shown in Additional file 1: Fig. S1C-I. **c**–**e** Comparisons between identified poly(A) sites and annotated poly(A) sites. The Y-axis represents the count of poly(A) sites, and the X-axis represent the distance between the identified poly(A) sites and the closest annotated poly(A) sites, **c** is for CEL-seq2/A dataset, **d** is for SCRB-seq/A dataset, and **e** is for Microwell-seq dataset. Additional examples are shown in Additional file 1: Fig. S2B, C. **f** Canonical poly(A) motif (AAUAAA) enrichments for novel poly(A) sites identified using five different scRNA-seq datasets, including CEL-seq2/A, CEL-seq2/B, SCRB-seq/A, SCRB-seq/B, and Microwell-seq. *P*-values and percentage of targets are shown. **g** The line plots illustrate the canonical poly(A) signal (AAUAAA) distribution from upstream 50 nt to downstream 50 nt to novel poly(A) sites. Y-axis: the canonical poly(A) signal (AAUAAA) frequency; X-axis: the distance from upstream 50 nt to downstream 50 nt to novel poly(A) sites. **h** The IGV plot depicts the read distributions on human CCDC173 gene. The upper three tracks represent the bulk RNA-seq read distributions of Gm12878, HepG2, and HEK293 cell line. The bottom track represents pooled scRNA-seq read distributions of HEK293 cell line. The identified novel poly(A) site is marked by the dashed red line

Currently available scRNA-seq protocols are developed from two main strategies, tag-based and full-length. The tag-based scRNA-seq methods with a designed unique molecular identifier (UMI) are either 3′-tag- or 5′-tag-based. It should be mentioned that 3-tag-based scRNA-seq methods are based on the strategy using oligo-dT priming to enrich the 3′-ends of transcripts, which are similar to several widely used high-throughput APA profiling methods. Based on the read coverage of 3′-tag-based scRNA-seq methods (Fig. 1a), we set out to explore whether this kind of scRNA-seq data could be applied to APA analysis.

To address this question, we collected five recently published 3′-tag-based scRNA-seq datasets, including two replicated CEL-Seq2 (cell expression by linear amplification and sequencing) datasets (A/B) for mouse embryonic stem cells (mESC), two replicated SCRB-seq (single-cell RNA barcoding and sequencing) datasets (A/B) for mESC and a Microwell-seq dataset for HEK293 cell line [27, 28]. In order to accurately evaluate the reliability of scRNA-seq data in poly(A) site identification, we first need to ensure that 3′-tag scRNA-seq methods indeed preferentially capture 3′-end of transcripts. We extracted the coordinates of 3′-ends from all aligned reads in scRNA-seq data and compared them with poly(A) sites annotated in two widely used poly(A) databases, including PolyA_DB 3 and GENCODE, respectively (Additional file 1: Fig. S1A-B) [29, 30]. The results suggested 3′-ends of scRNA-seq reads are enriched adjacent to annotated poly(A) sites, although different protocols exhibited distinct cumulative distributions (Additional file 1: Fig. S1A-B). To further evaluate the validity of these scRNA-seq data in poly(A) site identification, we pooled all aligned reads together and extracted reads containing poly(A) sequence (poly(A) reads) as poly(A) reads (see the “Methods” section). Notably, the poly(A) reads coverage decreased sharply around the annotated poly(A) sites to create peaks that could be used to infer the coordinates of poly(A) sites (Fig. 1b, Additional file 1: Fig. S1C-I).

According to these observations in 3′-tag-based scRNA-seq data, we referred to previous studies [20, 31] and developed a computational method that aim to de novo identify poly(A) sites using 3′-tag-based scRNA-seq data, regardless of any prior poly(A) sites annotation (Additional file 1: Fig. S2A). Firstly, we trimmed consecutive poly(A) sequences and tagged scRNA-seq reads into poly(A) and non-poly(A) reads. Then, we could obtain the genomic coordinates of 3′-ends of those tagged poly(A) reads and count the number of aggregated 3′-ends on each position from the aligned reads. The summits of clusters could be regarded as potential poly(A) sites. As the poly(A) reads may originate from internal poly(A) regions, we excluded those poly(A) sites adjacent to consecutive poly(A) sequences that were suspected to generate from internal priming. By further filtering those adjacent to annotated poly(A) sites, additional sites were regarded as novel poly(A) sites (Additional file 1: Fig. S2A).

Taking advantage of the collected 5 scRNA-seq datasets [27, 28], we set out to identify poly(A) sites for each dataset. To determine how well the poly(A) sites were identified using scRNA-seq data match annotated poly(A) sites, we calculate the distances from poly(A) sites identified using SAPAS to the closet annotated poly(A) sites for each scRNA-seq dataset. The results showed that the identified poly(A) sites exhibit a sharp peak around annotated poly(A) sites within 10 nt, suggesting that SAPAS could accurately identify the exact positions of annotated poly(A) sites using scRNA-seq data (Fig. 1c–e, Additional file 1: Fig. S2B-C).

In order to further evaluate the performance of poly(A) identification using SAPAS on scRNA-seq data, we conducted motif enrichment analysis on the novel poly(A) sites identified in each scRNA-seq dataset. The poly(A) signals are required for pre-mRNA cleavage and polyadenylation and usually found at approximately 15–30 nt upstream of the poly(A) sites. The canonical poly(A) signal is AAUAAA, which is predominant with greater than 50% frequency [3, 32]. The results of motif enrichment showed that the canonical poly(A) signal (AAUAAA) is top significantly enriched for each scRNA-seq dataset (Fig. 1f). Furthermore, the position-dependent frequency of the canonical poly(A) signal also illustrated that the novel poly(A) sites have the canonical poly(A) signal at the expected position, ~ 21 nucleotides upstream of poly(A) sites (Fig. 1g). These observations demonstrated the authenticity of poly(A) sites identified by SAPAS, indicating SAPAS could accurately identify the exact position of poly(A) sites. Additionally, two examples of novel identified poly(A) sites in mESC were shown in Additional file 1: Fig. S2D-E.

Moreover, novel intronic poly(A) site could also be identified using SAPAS. For example, a novel poly(A) sites (chr2:169686455-169686456:-) located in the first intron of coiled-coil domain containing 173 (CCDC173) was identified in the HEK293 scRNA-seq data, indicating that a truncated coding sequence was used in HEK293 cells (Fig. 1h). In addition, this intronic poly(A) site was also supported by bulk RNA-seq reads, but it was not reported in PolyA_DB 3 and GENCODE before [29, 30]. Interestingly, the intronic poly(A) site was used in a cell type-specific manner that it was mainly expressed in HEK293 cells that originally derived from human embryonic kidney cells, but other cells prefer to use the distal poly(A) sites, such as human GM12878 lymphoblastoid cells and HepG2 liver cancer cells (Fig. 1h). Together, these results demonstrated that 3′-tag-based scRNA-seq data could be used to identify poly(A) sites, allowing further exploration of APA in different cell types.

### Quantification of APA using 3′-tag-based scRNA-seq data

**Fig. 2:**
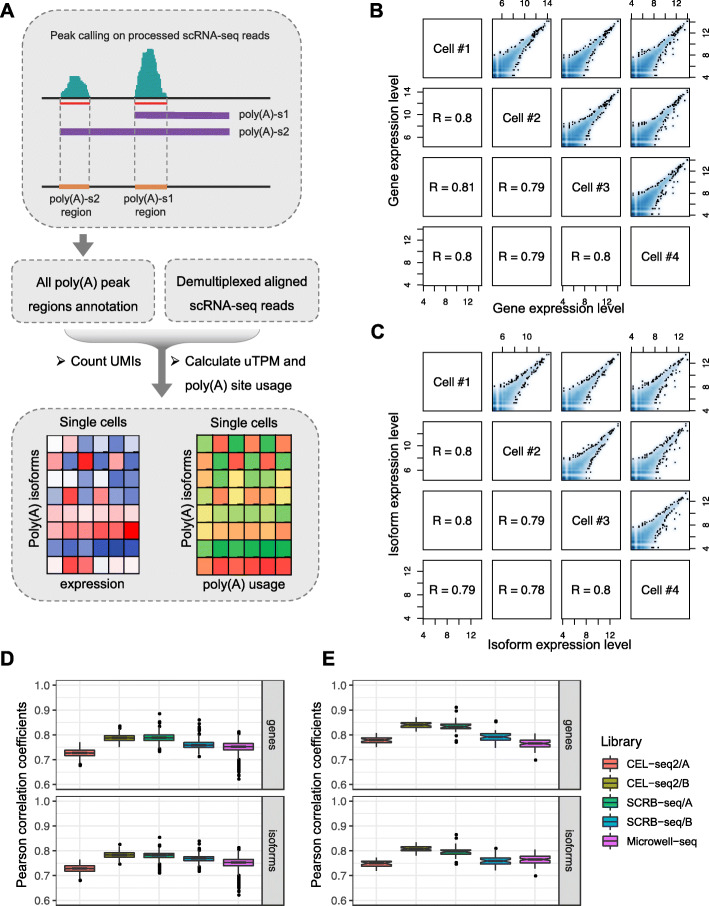
Quantification of poly(A) site usage using 3′-tag-based scRNA-seq data. **a** Schematic illustration of SAPAS to quantify poly(A) site usage using 3′-tag-based scRNA-seq data. **b** The smooth scatterplots pairwise comparing gene expression level of four randomly selected single cells from SCRB-seq/A dataset. The lower left indicates the Pearson correlation coefficients (R) for each comparison. **c** The smooth scatterplots pairwise comparing poly(A) isoform expression level of 4 randomly selected single cells from SCRB-seq/A dataset. The lower left indicates the Pearson correlation coefficients (R) for each comparison. **d** The boxplots depict the distributions of pairwise Pearson correlation coefficients of the expression level estimated in five different scRNA-seq datasets. The upper boxplot is for gene expression level and the lower boxplot is for poly(A) isoform expression level. Different datasets are represented in different colors as shown. **e** The boxplots depict the distributions of Pearson correlation coefficients of expression level between each single cell and pooled single-cell data. The upper boxplot is for gene expression level and the lower boxplot is for poly(A) isoform expression level. Different datasets are represented in different colors as shown

The pooled aligned reads of 3′-tag-based scRNA-seq data were clustered using the parametric clustering algorithm implemented in paraclu to identify the peak regions [33]. Combining defined poly(A) sites, we could assign peak regions to poly(A) sites for further quantification of APA. Once the genomic intervals of all poly(A) sites’ peak regions were identified, the transcript-level expression of distinct poly(A) isoforms could be estimated by counting reads aligned to each poly(A) site’s peak region for each single cell. Furthermore, to quantify the relative usage for each poly(A) site, we calculated the relative expression level of a specific poly(A) site isoform with respect to the total expression level of all poly(A) isoforms of the gene. Through this way, we could profile the poly(A) site usage at the single-cell level (Fig. 2a).

To assess the reproducibility and reliability of quantification of APA using SAPAS, we calculated the pairwise Pearson correlations of gene expression level and poly(A) isoform expression level across all single cells for each scRNA-seq dataset, respectively (Fig. 2b, c, Additional file 1: Fig. S3A-D, Additional file 1: Fig. S4A-D). The pairwise Pearson correlations of gene expression level were highly correlated that could reach about 0.8 and the correlations dropped slightly in isoform expression levels (Fig. 2d), suggesting that the estimation of poly(A) isoform expression level is reproducible as gene level, despite the fact that poly(A) isoforms only contained a fraction of the reads. One major challenge of scRNA-seq data analysis is the presence of dropout events where one gene is observed at a moderate or even high expression level in one cell but undetected in another cell, which is due to low amounts of RNA sequenced for each single cell [34]. To assess the effect of dropout events on quantification of APA, we calculated the Pearson correlations of gene expression level and poly(A) isoform expression level between each individual cell and artificial bulk sample constructed by simply summing the single-cell read counts, respectively (Fig. 2e). The marginal difference of Pearson correlations of poly(A) isoform expression level and gene expression level demonstrated that the dropout events do not introduce too many additional biases which could dramatically affect the accuracy of quantification of APA (Fig. 2e).

**Fig. 3:**
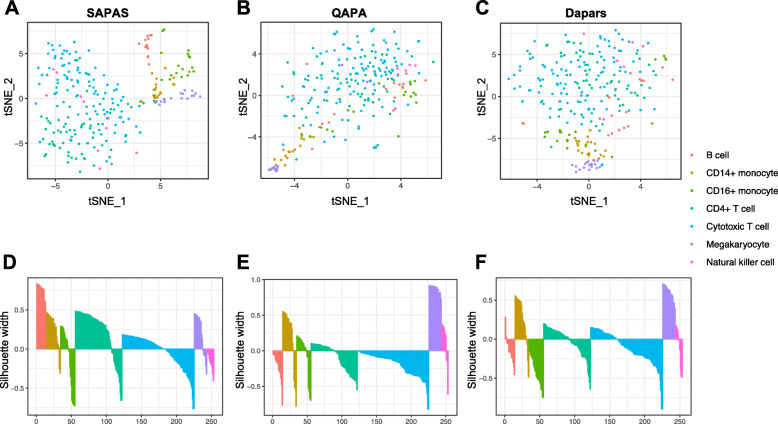
Benchmarking analysis of SAPAS on quantification of APA at single-cell level. **a**–**c** The t-SNE plots of single-cell APA profiles estimated by SAPAS (**a**), QAPA (**b**), and Dapars (**c**) on a benchmarking scRNA-seq dataset of PBMCs. The cell types are labeled using different colors as shown, including B cell, CD14+ monocyte, CD16+ monocyte, CD4+ monocyte, cytotoxic T cell, megakaryocyte, and natural killer cell. **d**–**f** The bar charts show the silhouette analysis of clustering results from single-cell APA profiles estimated by SAPAS (**d**), QAPA (**e**)**,** and Dapars (**f**). The Y-axis represents the silhouette width, and the X-axis represents single cells ordered by corresponding silhouette width decreasingly by cell types

To further illustrate the reliability of SAPAS, we also compared the APA profiles estimated from scRNA-seq data to those computed using bulk 3′-end sequencing data in HEK293 cells [21]. Despite the fact that the scRNA-seq data and bulk 3′-seq data were generated in different labs and using obviously different sequencing technologies, the estimated poly(A) isoform expression level correlated well between scRNA-seq data and bulk 3′-seq data (Pearson correlation R = 0.67, P < 2.2 × 10^−16^) (Additional file 1: Fig. S5A, B). In addition, to further demonstrate the performance of SAPAS on quantifying APA at the single-cell level, we applied SAPAS and other existing bioinformatics methods developed to analyze APA dynamics using conventional RNA-seq data, such as Dapars [13] and QAPA [22], to conduct benchmarking analysis on a CEL-seq2 dataset of peripheral blood mononuclear cells (PBMCs) [35]. The results of t-distributed stochastic neighbor embedding (t-SNE) showed that the single-cell APA profiles estimated by SAPAS could clearly separate single cells into different cell-type clusters, including B cells, T cells, monocytes, and megakaryocytes (Fig. 3a). Besides, several cell subtypes could also be revealed by single-cell APA profiles, such as CD4+ T cell and cytotoxic T cell (Fig. 3a). However, the single-cell APA profiles estimated using Dapars and QAPA could only cluster one or two cell types and failed to distinguish others (Fig. 3b, c). We also conducted silhouette analysis to quantitatively evaluate the clustering results. The silhouette width is widely used to quantitatively assess the quality of the clustering results. The observations showed that the silhouette widths of SAPAS are higher than Dapars and QAPA, indicating SAPAS outperform Dapars and QAPA on quantifying APA at the single-cell level using 3′-tag-based scRNA-seq data (Fig. 3d–f).

### SAPAS enables identification of novel poly(A) sites in GABAergic interneurons

We next employed SAPAS to study the genome-wide landscape of APA in phenotype-characterized GABAergic interneurons, using recently published 3′-tag-based scRNA-seq data generated from six non-overlapping GABAergic subpopulations with anatomical, physiological, and molecular evidence [36]. These GABAergic neurons include 64 CCK-positive basket cells (CCKC) [37], 132 chandelier cells (CHC) that innervate the axon initial segment of pyramidal neurons [25], 63 interneuron-selective cells (ISC) [38], 136 long-range-projecting GABAergic neurons (LPC) [39], 62 Martinotti cells (MNC) [40], and 127 fast-spiking parvalbumin-positive interneurons (PVBC) [41]. These individual neurons were manually sorted from micro-dissected motor and somatosensory cortexes of 6-week-old mice [36].

To systematically explore APA in these genetically labeled and phenotypically characterized GABAergic neurons, we first applied SAPAS to identify novel poly(A) sites for each GABAergic neuron type (Additional file 1: Fig. S6). As a result, several novel poly(A) sites were identified in each subtype (1,356 in CCKC, 1,016 in CHC, 620 in ISC, 961 in LPC, 674 in MNC, and 905 in PVBC, Additional file 1: Fig. S7A). Combining the novel poly(A) sites of each GABAergic neuron type, we altogether identified 3777 novel poly(A) sites in these GABAergic neurons. Among these combined novel poly(A) sites, 121 poly(A) sites were discovered in all 6 GABAergic neuron types (Additional file 1: Fig. S7A). Further motif analysis showed that the canonical poly(A) signal (AAUAAA) is top significantly enriched for each GABAergic neuron type (Additional file 1: Fig. S7B) and located at the expected position, ~ 21 nucleotides upstream of poly(A) sites, which is similar to the annotated poly(A) sites (Additional file 1: Fig. S7C). These observations demonstrated the reliability of these poly(A) sites in GABAergic neurons identified by SAPAS. As an example, across all GABAergic neuron types, a previously unannotated poly(A) site was identified in the 3′ UTR of the gene *Ran*, coding for a small GTP-binding protein that plays fundamental roles in regulating the translocation into and out of the cell nucleus [42] (Additional file 1: Fig. S7D). In addition, the canonical poly(A) motif (AAUAAA) was found ~ 20 nucleotides upstream of the poly(A) site (Additional file 1: Fig. S7D). Furthermore, to explore the potential underlying biological function of these novel poly(A) sites, we performed Gene Ontology (GO) enrichment analysis to assess whether these genes with novel poly(A) sites belong to specific GO terms. The enrichment results revealed that the novel poly(A) sites identified in different GABAergic neuron types are enriched for genes with synaptic communication-associated GO terms, such as presynaptic membrane, postsynaptic membrane, and axon part (Additional file 1: Fig. S8).

### APA profiles could be used to classify different GABAergic neuron types

Given that APA is known to be involved in numerous biological processes including development, cell differentiation, cell proliferation, and cell reprogramming [11–17], we next sought to investigate whether APA profiles could be used to determine GABAergic neuron identity. To address this question, we employed SAPAS to compute poly(A) site usage for all six different GABAergic neuron types. Taking the clustering result using gene expression level as reference, the t-SNE plots demonstrated that both poly(A) isoform expression and poly(A) site usage could also be used to separate different GABAergic neuron types clearly [43] (Additional file 1: Fig. S9).

**Fig. 4:**
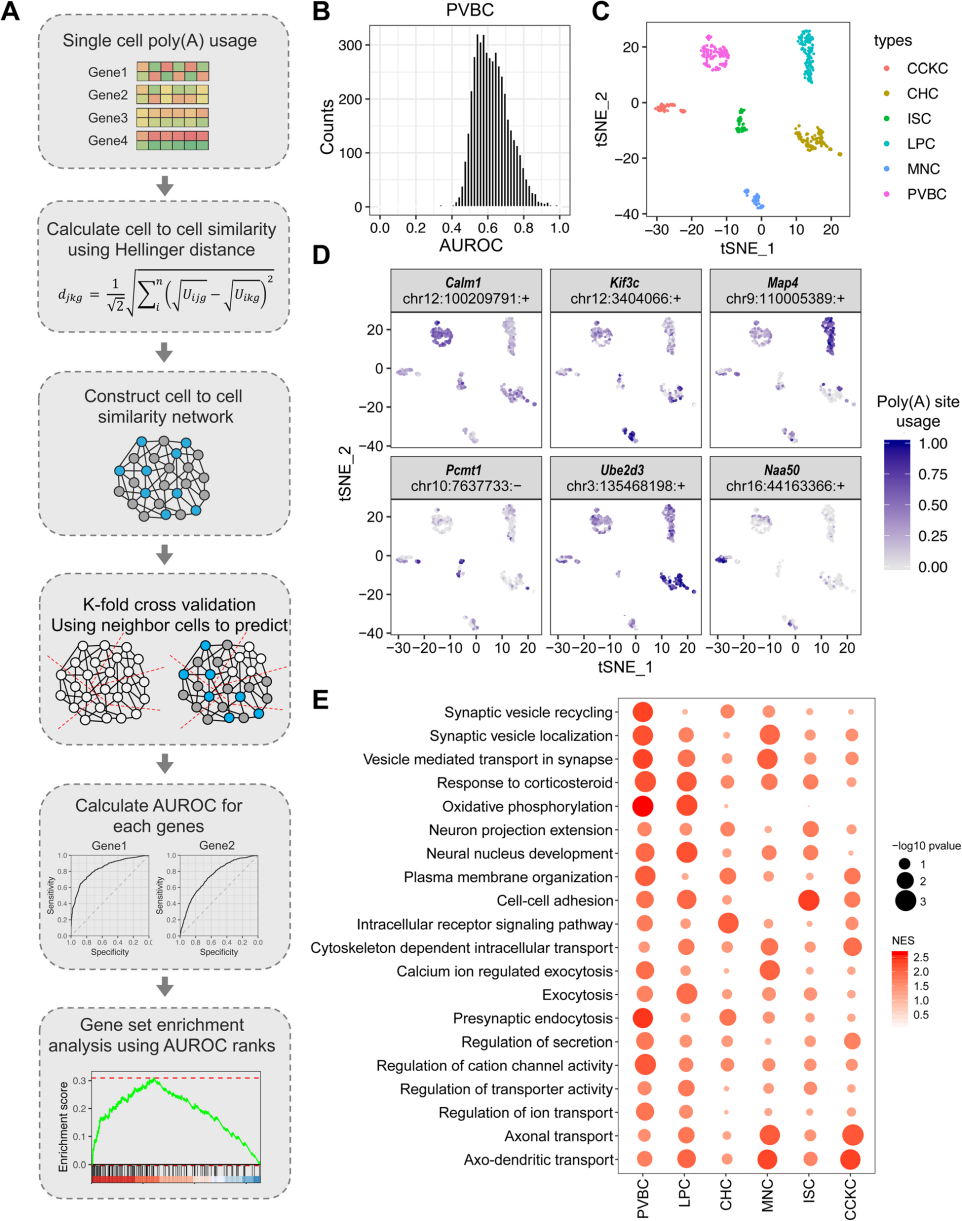
Cell type-specific APA events across GABAergic neurons. **a** Schematic overview of the machine learning-based method in SAPAS used to identify cell type-specific APA events. **b** The histogram depicts the distribution of AUROCs of all genes on classifying PVBC neurons from other GABAergic neuron types. The Y-axis represents the counts, and the X-axis represents the AUROC. **c** The t-SNE plot of six different GABAergic neuron types on gene expression level. The neuron types are labeled using different colors as shown. **d** The t-SNE plots depict the signal distributions of the poly(A) site usage of identified cell type-specific APA events for different GABAergic neuron types (*Calm1* for PVBC, *Kif3c* for MNC, *Map4* for LPC, *Pcmt1* for ISC, *Ube2d3* for CHC, and *Naa50* for CCKC). The blue gradient represents the poly(A) site usage as indicated. **e** Synaptic communication-related GO terms (biological process) enriched in genes with cell type-specific APA events for each GABAergic neuron type. The normalized enrichment scores (NES) calculated by GSEA are shown by red gradient, and the *P*-values are shown by circle size

Furthermore, in order to detect the cell type-specific APA events of GABAergic neuron types, we implemented a supervised machine learning-based method in SAPAS (Fig. 4a). The basic rationale was that single cell of the same cell type should exhibit a similar APA pattern for each gene. For each gene, we first calculate the similarity between all pairs of single cells based on the poly(A) site usage of this gene. In contrast to gene expression level, the poly(A) sites usage of one gene are not scalar, but vector. Thus, we used the Hellinger distance [44] commonly used to measure the similarity of two probability distributions to measure the similarity of cell pairs and construct the cell-to-cell similarity network. Next, we made use of the cross-validation strategy to randomly select some single cells as test set and others are training set. Then, we could predict cell types of the held-out test set by using a neighbor-voting algorithm based on the predefined cell-to-cell similarity network. Thus, the performance (area under the receiver operating characteristic (AUROC)) of separating one cell type from others using poly(A) site usage was calculated as cell-type specificity of APA events, and gene set enrichment analysis (GSEA) could be applied based on the AUROC ranks to probe potential functional associations (Fig. 4a).

Next, we have calculated the cell-type specificity of APA events in the GABAergic neuron dataset, and 4269 genes with multiple poly(A) sites were tested (Fig. 4b, Additional file 1: Fig. S10). In addition, we also performed differential usage analysis using one-versus-rest scheme for each GABAergic neuron type to detect significantly differential used poly(A) sites by constructing artificial bulk data from single-cell data. The observations showed that the genes with significantly differential used poly(A) sites rank top on AUROC in each neuron type (Additional file 1: Fig. S10). By setting a threshold on AUROC (AUROC > 0.8), we could define a set of genes with cell type-specific APA events for each neuron type. Several genes with cell type-specific APA events were shown in Fig. 4c, d. For example, the gene coding for Calmodulin 1 (*Calm1*), a key integrator of calcium signaling that is involved in guiding axon projections to create connections with other neurons or tissues [45, 46], predominantly uses the distal poly(A) site (chr12:100209791:+) in PVBCs compared with other GABAergic neuron types.

Furthermore, we performed GSEA to assess whether genes with cell type-specific APA events belong to specific biological functions or pathways. The results showed that those genes with higher cell-type specificity were significantly enriched in synaptic connectivity and input-output signaling-related function categories, including synaptic vesicle release machinery, cell adhesion molecules, ion channels, and intracellular receptor signaling (Fig. 4e). In addition, GSEA using cellular component gene sets suggested that these genes encode proteins that localize along or close to the cell or synaptic membrane, such as presynapse, postsynapse, and vesicle membrane (Additional file 1: Fig. S11). Collectively, these observations suggested that the anatomical and physiological differences across different GABAergic neuron types may in part be mediated by APA of genes involved in synaptic communication.

Given that the GABAergic neuronal identity is reported to be encoded in functionally congruent gene expression [36], we next sought to investigate whether the cell-type specificity of APA events is primarily due to gene expression specificity. To address this question, we applied the EWCE method to calculate the cell-type expression specificity for each gene in each GABAergic neuron type (Additional file 1: Fig. S12), which is a metric that represents the proportion of expression of one gene found specifically in one cell type compared to all cell types [47, 48]. We plotted the gene expression specificity of gene sets with cell type-specific APA events for each GABAergic neuron type (Additional file 1: Fig. S13). The results showed that the large majority (> 80%) of genes with cell type-specific APA do not display high cell-type expression specificity, suggesting that the APA profiles difference could be another source of heterogeneity across different GABAergic neuron types, partially independent of gene expression level.

### Cell type-specific APA events alter 3′ UTR length and CDS of genes

**Fig. 5:**
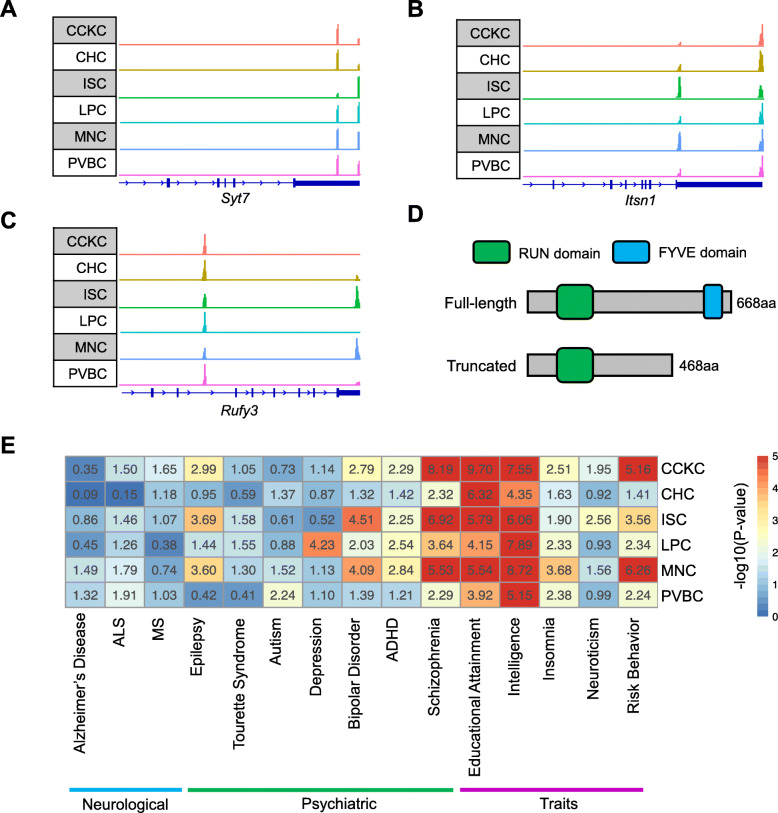
3′ UTR and CDS altered by cell type-specific APA events. **a**, **b** Examples of cell type-specific APA events of ISC that could alter the 3′ UTR length. Pooled scRNA-seq read coverages around poly(A) sites are displayed using IGV. Each row represents a specific GABAergic neuron type. **a** distal poly(A) site of *Syt7* is predominantly used in ISC, leading to a longer 3′ UTR. **b** proximal poly(A) site of *Itsn1* is predominantly used in ISC, leading to a shorter 3′ UTR. **c** An example of cell type-specific APA events that could alter CDS. The intronic poly(A) site located in the 12th intron of *Rufy3* is predominantly used in CCKC, CHC, LPC, and PVBC, leading to a truncated CDS, but ISC and MNC prefer to use the distal poly(A) site. Pooled scRNA-seq read coverages around poly(A) sites of *Rufy3* are displayed, and each row represents a specific GABAergic neuron type. **d** Difference between the protein product generated from full-length and truncated *Rufy3* isoforms. The truncated protein of *Rufy3* lacks a FYVE-related domain. **e** The heatmap depicts the LDSC results of genetic variants associated with brain-related diseases and quantitative traits for each GABAergic neuron type. The significance of enrichment for the altered regions of cell type-specific APA events is displayed as -log_10_(*P*-value)

In addition to 3′ UTR length difference, the coding sequence (CDS) could also be affected by APA located in upstream introns and internal exons in about 40% of mammalian genes [49, 50]. To explore the biological functions of cell type-specific APA events, we assessed potential 3′ UTR or CDS changes due to cell type-specific APA events for each GABAergic neuron type. Most cell type-specific APA events resulted in 3′ UTR length difference only, without impact on CDS. For example, *Syt7* coding for Synaptotagmin-7, brain-specific calcium-dependent proteins which have been shown to regulate synaptic exocytosis and neurotransmitter release [51, 52], predominantly used the distal poly(A) sites in ISC compared to other types of GABAergic neurons (Fig. 5a). Conversely, *Itsn1*, a multidomain scaffolding and adaptor protein involved in the synaptic vesicle, predominantly expressed the short 3′ UTR APA isoforms in ISC, whereas other GABAergic neurons expressed long 3′ UTR APA isoforms (Fig. 5b). In addition, cell type-specific APA events with impact on the CDS were also found in different GABAergic neuron types. For instance, *Rufy3*, a neuronally enriched protein which has been implicated in regulating the generation of neuronal polarity formation and axon growth [53, 54], mostly expressed the full-length isoform in ISC and MNC, whereas an intronic poly(A) site of *Rufy3* was predominantly used in other types of GABAergic neurons, resulting in the APA isoform with a truncated CDS (Fig. 5c). In further, we found that the truncated protein of *Rufy3* generated by intronic polyadenylation lacks the FYVE-related domain compared to the full-length protein (Fig. 5d). Notably, the FYVE-related domain is an evolutionarily conserved domain which could bind with high specificity to phosphatidylinositol 3-phosphate (PI(3)P) to localize proteins to endosomes [55, 56]. In addition, a recent study demonstrated that *Rufy3* is essential for caspase-mediated axon degeneration [57]. Collectively, the result suggested that the intracellular localization and biological function of *Rufy3* protein may be altered by APA in different GABAergic neuron types. In addition, to further illustrate the cell-type specificity of these APA events, we collected an independent public scRNA-seq dataset of mouse neocortex that containing GABAergic interneurons [58]. This dataset is generated by Smart-seq2 method which is a full-length scRNA-seq method. We could extract those single cells with cell types corresponded to the GABAergic neuron type in our study (Additional file 1: Fig. S14A, B). Then, we exploited QAPA, which are suitable for full-length RNA-seq data, to compute the single-cell APA profiles. The observations showed that the APA patterns of *Syt7*, *Itsn1*, and *Rufy3* among different neuron types are in agreement with previous results, demonstrating the authenticity of cell type-specific APA events identified by SAPAS in our study (Additional file 1: Fig. S14C-H).

To further explore the potential functional consequences of altered 3′ UTR or CDS of APA, we sought to intersect GWAS signals with cell type-specific APA events with the goal of systematically linking particular diseases or traits to APA. Given a large faction of APA events are quite well conserved between human and mouse [29], we applied the linkage disequilibrium (LD) score regression (LDSC) method to quantify the enrichment of heritability for human traits and diseases within altered 3′ UTR or CDS of cell type-specific APA events [59]. To do so, we first lifted over human SNPs to orthologous coordinates in mouse genome and then calculated the enrichment of heritability for 15 brain-related diseases and quantitative traits, including Alzheimer’s disease, amyotrophic lateral sclerosis (ALS), multiple sclerosis (MS), epilepsy, Tourette syndrome, autism, depression, bipolar disorder, attention deficit hyperactivity disorder (ADHD), schizophrenia, educational attainment, intelligence, insomnia, neuroticism, and risky behavior (Fig. 5e). As a result, we observed statistically significant enrichments for heritability for psychiatric disorders and brain traits, such as schizophrenia, ADHD, bipolar disorder, and educational attainment (Fig. 5e). In previous studies, converging evidence suggests that dysfunction of GABAergic interneurons is critical for basic neural circuit function whose dysfunction is linked to the pathophysiology of several psychiatric diseases [60, 61]. Taken together, these results suggested that cell type-specific APA events were involved in defining the physiological properties of GABAergic neurons.

### Bimodality of APA could reveal subpopulations of chandelier cells

**Fig. 6:**
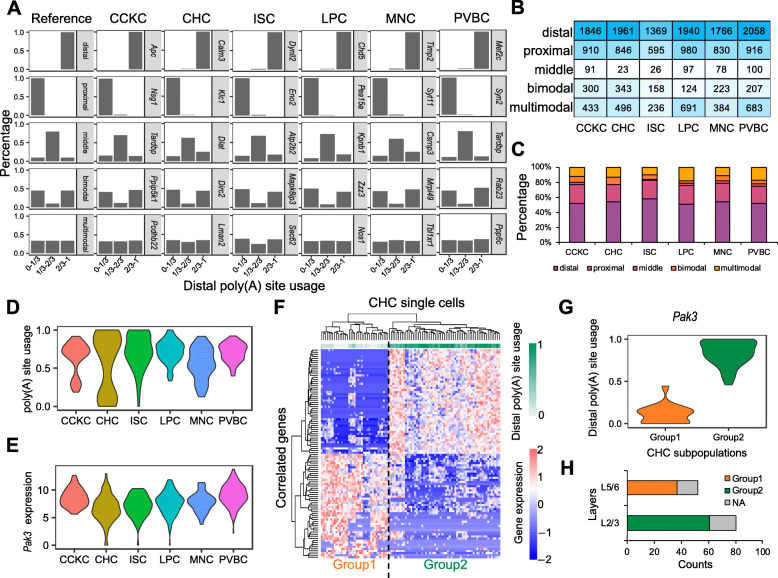
Modality of poly(A) site usage in GABAergic neurons. **a** The distributions of poly(A) site usage for five different modalities, including distal, proximal, middle, bimodal, and multimodal. The leftmost column shows the reference distributions for modality assignment. Examples of genes with different modalities in CCKC, CHC, ISC, LPC, MNC, and PVBC are shown. B, C The table (**b**) depicts the numbers of genes with different modalities for each GABAergic neuron type. Accordingly, the stacked bar chart in **c** depicts the proportion of genes with different modalities. **d** The violin plots show the distributions of distal poly(A) site usage of *Pak3* in different GABAergic neuron types. **e** The violin plots show the distribution of *Pak3* gene expression level in different GABAergic neuron types. **f** Bimodality of distal poly(A) site usage of *Pak3* could reveal different subpopulations of CHCs. The heatmap depicts the genes top correlated with distal poly(A) site usage of *Pak3* cluster CHCs into two groups (Group1 vs Group2). The columns represent CHC single cells, and the rows represent correlated genes. The gradient blue to red represents gene expression level in log_2_(uTPM + 1). The gradient green represents distal poly(A) site usage of *Pak3*. **g** The violin plot shows the distributions of distal poly(A) site usage of *Pak3* in Group1 and Group2 CHCs. **h** The stacked bar chart depicts the proportions of Group1 and Group2 single cells of CHCs in upper (L2/3) and deeper (L5/6) cortical layers. NA is for those single cells in which poly(A) site usage of *Pak3* are not detected

Quantification of APA at the single-cell level provides us an opportunity to study the cell-to-cell variability of APA. To categorize the distributions of poly(A) site usage at the single-cell level, we implemented a method to classify each gene into one of five modalities, including (1) distal, where the distal poly(A) sites were predominantly used in the majority of cells; (2) proximal, where the proximal poly(A) sites were predominantly used in most cells; (3) bimodal, where two subpopulations of cells with either distal poly(A) sites or proximal poly(A) sites were used; (4) middle, where the moderate usage of distal poly(A) sites can be observed; and (5) multimodal, where distal poly(A) site usages were random from 0 to 1 (Fig. 6a). We binned poly(A) site usage into [0~1/3, 1/3~2/3, 2/3~1] and set binned distributions for different modalities as reference (Fig. 6a). Through computing the distance measured by Jensen-Shannon divergence between each gene’s binned distribution of distal poly(A) site usage and reference binned distributions, the modality of reference binned distribution with the smallest distance was assigned. Thus, we designated genes into different modalities for each GABAergic neuron type (Fig. 6a). For instance, the gene *Tardbp* with critical roles in splicing in neurons exhibits the middle modality of distal poly(A) site usage in CCKC. In all six GABAergic neuron types, genes within distal modality account for more than 50% of all genes analyzed (Fig. 6b, c), which lines up with previous studies that distal poly(A) sites are favored in nervous systems, resulting in isoforms with longer 3′ UTRs [62, 63]. Besides, genes that exhibit proximal modality account for ~ 25% of all genes, whereas bimodality and multimodality account for ~ 10% and ~ 15%, respectively (Fig. 6b, c).

As genes within bimodality exhibited higher variability of distal poly(A) site usage compared with other modalities (Additional file 1: Fig. S15), we surmised that they could be used to identify subpopulations from a specific GABAergic neuron type. As an example, we observed that the gene *Pak3* (p21-activating kinase 3) exhibited bimodality of distal poly(A) site usage in CHCs (Fig. 6d, e). *Pak3* is a serine/threonine kinase preferentially expressed in neurons that functions as a downstream effector of the Rho family of GTPase and plays a critical role in regulating neurite growth, as well as synapse formation and plasticity [64–66]. CHCs could be separated into two groups (Group1 and Group2) by distal poly(A) site usage of *Pak3* (Fig. 6f). In addition, we identified genes that top correlated and anticorrelated with distal poly(A) site usage of *Pak3*, which also clustered the CHCs into two groups (Fig. 6f). We observed that the distal poly(A) site was predominantly used in Group2 CHCs compared to Group1 CHCs (Fig. 6f, g). Notably, we found that all of the Group1 CHCs were obtained from deeper layers (L5/6), whereas Group2 CHCs were from upper layers (L2/3) (Fig. 6h), which was consistent with previous studies showing CHCs in distinct layers are different subgroups that are recruited by distinct cortical inputs and regulate different populations of pyramidal neurons [25]. Besides, several additional examples with the potential to subtype GABAergic neurons were also found, such as *Ythdf3*, *Dicer1*, *Efr3a*, and *Cbx5* (Additional file 1: Fig. S16). Collectively, these results demonstrated that the modalities estimated from quantification of APA at the single-cell level provide us an additional layer of information to reveal cell subpopulations.

## Discussion

In this study, we presented a computational approach, SAPAS, to conduct APA analysis using 3′-tag-based scRNA-seq data. First, making use of several published 3′-tag-based scRNA-seq datasets utilizing different protocols in different cell lines, we have provided multiple lines of evidence to reveal the reliability and validity of single-cell APA analysis using SAPAS. These results demonstrated the potential of using scRNA-seq data to characterize APA, although these data were intentionally generated to quantify gene expression level at a single-cell level. Furthermore, we have applied SAPAS to a comprehensive scRNA-seq dataset of six different GABAergic neuron types. From the dataset, 3777 poly(A) sites not annotated before were identified to expand the poly(A) site repository of the mouse. Moreover, based on the quantification of APA at the single-cell level, we discovered that the poly(A) site usage exhibits a cell type-specific manner. To better understand APA in GABAergic neurons, a machine learning-based method in SAPAS was used to identify cell type-specific APA events for each neuron type. GSEA enabled us to discover that these cell type-specific APA events are enriched for genes related to synaptic architecture and communications. Further integrative analysis with GWAS data demonstrated that 3′ UTR or CDS differences derived from cell type-specific APA events were involved in regulating the physiological function of GABAergic neurons. Finally, taking advantage of single-cell methods, we could classify APA patterns into five categories, including distal, proximal, middle, bimodal, and multimodal. As demonstrated in this study, the bimodality of APA at the single-cell level could demarcate cell subpopulations.

An inherent limitation of SAPAS in detecting novel poly(A) sites using 3′-tag-based scRNA-seq data is that it could lead to the identification of artifactual poly(A) sites due to internal priming events. 3′-tag-based scRNA-seq methods have relied on priming with oligo-dT containing primers for library construction. However, the oligo-dT priming could occur in internal homo-polymeric stretches of adenines, leading to the identification of artifactual poly(A) sites. To address this question, we have applied a heuristic filters method to removing potential internal priming events based on the arbitrary cutoff of the number of adenines in the genome sequences. However, this could lead to unavoidably excluding some true poly(A) sites or including some artifactual poly(A) sites. In addition, we could integrate a machine-learning method that utilizing more sequence features around poly(A) sites to exclude internal priming events, in order to further increase the accuracy of SAPAS in the identification of poly(A) sites. Another limitation of SAPAS is that the accuracy of quantification of APA is influenced by sequencing depth of scRNA-seq libraries. If the sequencing depth of scRNA-seq libraries is too low, the poly(A) isoforms may not be sampled adequately, leading to inaccurate quantification of APA at the single-cell level. Therefore, we have to filter those single cells with low sequencing depth and could only capture APA profiles for genes with enough UMI counts but not for genes with low abundance.

By applying SAPAS to the scRNA-seq data of GABAergic neurons, a comparison of APA profiles of different GABAergic neuron types reveals cell type-specific APA events. Through GSEA, we observed enrichments for synaptic communication-related genes among those genes with cell type-specific APA events, suggesting another layer of regulation of neuronal identities and properties. This study further discovered that cell type-specific APA events could not only alter 3′ UTR, which potentially affect miRNA- or RBP-based regulation, but also alter the coding sequence, leading to different protein products. Furthermore, to bridge the gap between cell type-specific APA patterns and biological functions, we combined the GWAS data to calculate the enrichment of heritability for brain-related diseases and traits. The observations showed that the altered gene regions by cell type-specific APA were significantly associated with several psychiatric diseases, such as schizophrenia, ADHD, and bipolar disorder, suggesting potential links between APA and psychiatric-related functions. Besides, the cell type-specific APA were also associated with several cognitive traits, such as educational attainment. These results are in agreement with previous studies that GABAergic neurons are implicated in cognitive functions. GABAergic interneurons are the main inhibitory neurons that modulate excitatory signals, which are critical to cognitive function-related neural oscillation and information integration and processing [67–69]. Dysfunctional GABAergic activity could disrupt the excitatory/inhibitory balance in the cortex, leading to impaired neural oscillations underlying cognitive dysfunction [70]. These observations raised the possibility that APA events are implicated in cell identity of GABAergic neurons and play important roles in neural circuit formation. However, the underlying mechanisms of these APA events in cellular properties or function maintenance should be further experimentally validated in the future.

Finally, the scRNA-seq data of GABAergic neurons provides us an opportunity to assess the cell-to-cell modality of APA for each neuron type. The results showed that the distal modality is the predominant APA pattern in different GABAergic neurons, which is consistent with previous reports that long 3′ UTRs are favored in neurons [62, 63]. Interestingly, we found that the bimodal APA patterns of several genes could be used to subtype GABAergic neuron types. For instance, we have shown that *Pak3* exhibits a bimodal APA pattern in CHCs, which could be used to classify CHCs into two groups from different laminar positions, upper and deeper layers. Therefore, further studies of these results could expand our understanding of the molecular genetic basis of GABAergic neuron types.

## Conclusions

In this study, we developed and applied SAPAS, a new method that quantitatively infers APA at the single-cell level from scRNA-seq data. Application of SAPAS reveals cell type-specific APA events across different GABAergic neuron types. Significant enrichments of heritability for several psychiatric disorders and brain traits were observed in cell type-specific APA events. Also, as demonstrated in this study, the bimodal APA events could demarcate cell subpopulations. SAPAS thus enabled systematic APA characterization at the single-cell level, expanding our understanding of APA by leveraging the wealth of existing scRNA-seq data.

## Methods

### Systematic Alternative Polyadenylation Analysis at Single-cell level (SAPAS)

#### Data preprocessing of 3′-tag-based scRNA-seq data

For each 3′-tag-based scRNA-seq dataset, we first extracted the cell barcode and UMI from read1 and added them to read name to label the read2. Then, the labeled read2 were processed by trimming consecutive poly(A)s (A = 8), and those trimmed reads shorter than 20 bp were discarded. Besides, the “polyA” or “non-polyA” tag was added to each read name based on whether consecutive poly(A) located in the read sequence. Then, these processed reads were aligned to the reference genome using HISAT2 with default settings [71]. The reference genomes are downloaded from ENSEMBL, including the GRCh38 (hg38) for humans and the GRCm38 (mm10) for mice. Uniquely mapped reads were extracted, and PCR duplicates were removed using the cell barcode, UMI, and the aligned read end coordinate. Finally, the aligned reads could be split to each single cell in bam format by demultiplexing reads using the cell barcode.

#### Identification of poly(A) sites using scRNA-seq data

For each cell type, we pooled the aligned reads together and retrieved poly(A)-containing reads based on the “polyA” tags in read name. From these poly(A)-containing reads, those potential internal primed reads were removed using a heuristic method that reads with six or more consecutive adenines in the 20 bp immediately downstream from the reads’ end in the genomic sequence. Then, we got the 3′-end for each filtered poly(A)-containing read and retained those 3′-ends located in 3′ UTR regions defined from GENCODE annotations for human and mouse genes (GENCODE releases v28 and vM16, respectively) as poly(A) tags [30]. To call the poly(A) site, these poly(A) tags located within short distance (20 bp) of each other were clustered using distance clustering algorithm. The poly(A) tag cluster regions were further filtered by setting a threshold on normalized poly(A) tag counts. Finally, the summits of the filtered cluster regions were assigned as poly(A) sites.

#### Quantification of poly(A) site usage using scRNA-seq data

First, combining the novel identified poly(A) sites and annotated poly(A) sites defined in polyA_DB 3 [29], we could compile a comprehensive poly(A) sites set. Then, the pooled aligned reads of each cell type were clustered using parametric clustering algorithm to identify peak regions [33]. The peak regions across different cell types were merged using bedtools *merge* [72]. By intersecting with gene bodies defined from GENCODE annotations, only peak regions overlapped with gene bodies were remained for further analysis. The retained peaks regions could be assigned to previously compiled poly(A) sites set. Then, using the genomic intervals of peak regions assigned to poly(A) sites, we could count total reads aligned to each peak region for each gene at the single-cell level. Furthermore, to quantify the relative usage of poly(A) sites, we calculated the relative expression level of a specific poly(A) site isoform over the total expression level of all poly(A) isoforms of the gene, defined by ${U}_{ig}=\frac{C_{ig}}{\sum_i^n{C}_{ig}}$, where *g* is a given gene, *C*_*ig*_ is the UMI counts of poly(A) isoform *i* in gene *g*, and *n* is the number of poly(A) isoforms of the gene. To avoid zeros in the denominator, poly(A) site usage was only calculated for the genes detected. Through this way, we could profile the poly(A) site usage at the single-cell level for each scRNA-seq dataset.

#### Identification of cell type-specific APA events

To measure the cell-type specificity of APA events, we designed a supervised machine learning-based method. For each APA gene, we first computed the pairwise similarity of APA to construct the cell-to-cell similarity network. The poly(A) site usage is defined by ${U}_{ijg}=\frac{C_{ijg}}{\sum_i^n{C}_{ijg}}$, where *g* is a given gene, *j* is a given single cell, *C*_*ijg*_ is the UMI counts of poly(A) isoform *i* of gene *g* in single cell *j*, and *n* is the number of poly(A) isoforms of the gene. Because the sum of poly(A) sites usage is equal to one for any specific gene, a gene with *n* poly(A) isoforms in a specific single cell could be represented as a point in a *n*-dimensional space, where the coordinates are the poly(A) site usages. In addition, we could measure the distance using Hellinger distance between two single cells for any specific gene in this *n*-dimensional space. Thus, the Hellinger distance between single cell *j* and *k* is defined by : ${d}_{jkg}=\frac{1}{\sqrt{2}}\sqrt{\sum_i^n{\left(\sqrt{U_{ijg}}-\sqrt{U_{ikg}}\right)}^2}$, and the similarity between single cell *j* and *k* is defined by : *S*_*jkg*_ = 1 − *d*_*jkg*_ as Hellinger distance is naturally between 0 and 1. Pairwise similarity of all single cells could be calculated to construct the cell-to-cell similarity network. Next, we employed the cross-validation strategy to randomly separate all single cells into training and test sets. Then a neighbor-voting algorithm was employed to predict cell types of single cells in test sets based on their similarity to single cells in training sets. Thus, the mean AUROC for cross-validation was calculated for each gene.

### Benchmark analysis of SAPAS

To illustrate the performance of SAPAS on quantifying single-cell APA profiles, we applied SAPAS, DaPars [13], and QAPA [22] to a CEL-seq2 dataset of PBMCs to conduct benchmark analysis [35]. This scRNA-seq dataset was downloaded from Gene Expression Omnibus (GEO) under accession number GSE132044 [35]. The single-cell APA profiles estimated by SAPAS, Dapars, and QAPA were then used for clustering and visualized by t-SNE. Then, we conducted a silhouette analysis to compute the silhouette width using the *silhouette* function of R package *cluster.*

### APA analysis of the scRNA-seq data of GABAergic neurons

We applied SAPAS to the scRNA-seq data of six different GABAergic neuron types [36]. Novel poly(A) site sets were first identified for each neuron type separately. Then, the final novel poly(A) site set was identified by merging the novel poly(A) sites form different neuron types using bedtools *merge* [72]. Next, SAPAS was used to quantify poly(A) site usage for each single cell in this scRNA-seq dataset. Genes were filtered to be detected in at least 10 single cells of a given neuron type for following analyses, including identification of cell type-specific APA events and estimation of modality of poly(A) site usage.

### Calculation of enrichment of heritability

We adapted the method in Cusanovich et al.’s study [73] to compute the enrichment of heritability for brain-related diseases or traits within the altered regions of cell type-specific APA events. First, we lifted over all the SNPs used in LDSC software from human genome to mouse genome (https://github.com/bulik/ldsc) [74]. We then obtained full summary statistics for GWAS studies on Alzheimer disease, autism, depression, bipolar disorder, schizophrenia, and neuroticism from Broad LD Hub (https://data.broadinstitute.org/alkesgroup/sumstats_formatted). Additional summary statistics for GWAS on multiple sclerosis [75], amyotrophic lateral sclerosis (ALS) [76], Parkinson’s disease [77], epilepsy [78], attention deficit hyperactivity disorder (ADHD) [79], insomnia [80], educational attainment [81], intelligence [82], and risky behavior [83] were downloaded separately. Then, we calculated the enrichments of heritability within the altered regions of cell type-specific APA events using LDSC according to the recommended workflow.

### Estimation of modality of poly(A) site usage

For each gene, we divided the distal poly(A) site usage into three parts [0~1/3, 1/3~2/3, 2/3~1] and counted the percentage for each part. Taking reference distributions for five different modalities, including distal, proximal, middle, bimodal, and multimodal (Fig. 6a), we could calculate Jensen-Shannon divergence between the percentage and reference distributions [84]. Finally, we selected the modality of the closest reference distribution as the modality of the gene’s poly(A) site usage.

### Identification of genes that  correlate with APA events

To identify the genes that correlate with bimodal APA events, we first normalized the absolute UMI counts of each gene to the total unique UMI counts across all genes in each single cell in order to calculate the unique transcripts per million (uTPM) as normalized expression level. Then, we calculated the Pearson correlations between all genes’ normalized expression level and distal poly(A) site usage of the given gene. Genes with Pearson correlations that rank top 50 correlated and anticorrelated were regarded as correlated genes. Then, the correlated genes were subsequently used to cluster single cells by hierarchical clustering method. The heatmap was plotted using *aheatmap* function of R package *NMF*.

## Supplementary Information

**Additional file 1:** **Figure S1-S17. Fig. S1.** The distributions of poly(A) reads around poly(A) sites annotated in GENOCDE and PolyA_DB_3 in 3’-tag-based scRNA-seq data. **Fig. S2.** Identification of poly(A) sites using 3’-tag-based scRNA-seq data. **Fig. S3.** Pairwise comparisons of gene expression level and poly(A) isoform expression level for CEL-seq2. **Fig. S4.** Pairwise comparisons of gene expression level and poly(A) isoform expression level for SCRB-seq and Microwell-seq. **Fig. S5.** Comparisons of gene expression level and ply(A) isoform expression level estimated from Microwell-seq and 3’-seq data. **Fig. S6.** The distributions of poly(A) reads around poly(A) sites annotated in GENCODE in scRNA-seq data of GABAergic neurons. **Fig. S7.** Novel poly(A) sites in GABAergic neurons. **Fig. S8.** GO enrichment for genes with novel poly(A) sites. **Fig. S9.** Clustering of GABAergic neurons. **Fig. S10.** Comparisons of SAPAS and differential poly(A) usage analysis. **Fig. S11.** GO terms enriched in genes with cell-type specific APA events for each GABAergic neuron type. **Fig. S12.** Cell-type expression specificity for each gene in different GABAergic neuron types. **Fig. S13.** Cell-type expression specificity for genes with cell-type specific APA events. **Fig. S14.** Validation of the cell-type specific APA events using a Smart-seq2 dataset from Tasic et al. study. **Fig. S15.** Mean-variance relations of distal poly(A) site usage in different GABAergic neuron types. **Fig. S16.** Examples with the potential to demarcate subpopulations of GABAergic neurons.

## Data Availability

The scRNA-seq datasets of mESCs by CEL-seq2 and SCRB-seq method used in this study were downloaded from NCBI GEO (https://www.ncbi.nlm.nih.gov/geo/) under accession number GSE75790 [27, 85], including two replicates (A/B) for each method. The scRNA-seq dataset of HEK293 cell line by Microwell-seq method was downloaded from GEO under accession number GSE108097 [28, 86]. The 3′-seq dataset of HEK293 cell line was downloaded from the NCBI Sequence Read Archive (https://www.ncbi.nlm.nih.gov/sra) under accession number SRP029953 [21, 87]. The scRNA-seq dataset of six different GABAergic neuron types was downloaded from GEO under accession number GSE92522 [36, 88]. The code for SAPAS is available at https://github.com/YY-TMU/SAPAS.

## Abbreviations

APA: Alternative polyadenylation
3′ UTR: 3′ untranslated regions
scRNA-seq: Single-cell RNA-seq
CHC: Chandelier cell
UMI: Unique molecular identifier
mESC: Mouse embryonic stem cells
PBMC: Peripheral blood mononuclear cell
t-SNE: t-distributed stochastic neighbor embedding
CCKC: CCK-positive basket cell
ISC: Interneuron-selective cell
LPC: Long-range-projecting GABAergic 228 neuron
MNC: Martinotti cell
PVBC: Fast-spiking parvalbumin-positive 229 interneuron
AUROC: Area under the receiver operating characteristic
GSEA: Gene set enrichment analysis
CDS: Coding sequence
LDSC: Linkage disequilibrium score regression
ALS: Amyotrophic lateral sclerosis
MS: Multiple sclerosis
ADHD: Attention-deficit hyperactivity disorder
uTPM: Unique transcripts per million
