# Supplementary material for: Single-cell alternative polyadenylation analysis delineates GABAergic neuron types
Source: BMC Biol. 2021 Jul 23;19:144. doi: 10.1186/s12915-021-01076-3 (PMC8299648; doi:10.1186/s12915-021-01076-3)
Supplement: Supplementary file 1 — Additional file 1: Figure S1-S17. Fig. S1. The distributions of poly(A) reads around poly(A) sites annotated in GENOCDE and PolyA_DB_3 in 3’-tag-based scRNA-seq data. Fig. S2. Identification of poly(A) sites using 3’-tag-based scRNA-seq data. Fig. S3. Pairwise comparisons of gene expression level and poly(A) isoform expression level for CEL-seq2. Fig. S4. Pairwise comparisons of gene expression level and poly(A) isoform expression level for SCRB-seq and Microwell-seq. Fig. S5. Comparisons of gene expression level and ply(A) isoform expression level estimated from Microwell-seq and 3’-seq data. Fig. S6. The distributions of poly(A) reads around poly(A) sites annotated in GENCODE in scRNA-seq data of GABAergic neurons. Fig. S7. Novel poly(A) sites in GABAergic neurons. Fig. S8. GO enrichment for genes with novel poly(A) sites. Fig. S9. Clustering of GABAergic neurons. Fig. S10. Comparisons of SAPAS and differential poly(A) usage analysis. Fig. S11. GO terms enriched in genes with cell-type specific APA events for each GABAergic neuron type. Fig. S12. Cell-type expression specificity for each gene in different GABAergic neuron types. Fig. S13. Cell-type expression specificity for genes with cell-type specific APA events. Fig. S14. Validation of the cell-type specific APA events using a Smart-seq2 dataset from Tasic et al. study. Fig. S15. Mean-variance relations of distal poly(A) site usage in different GABAergic neuron types. Fig. S16. Examples with the potential to demarcate subpopulations of GABAergic neurons. [file 12915_2021_1076_MOESM1_ESM.pdf]

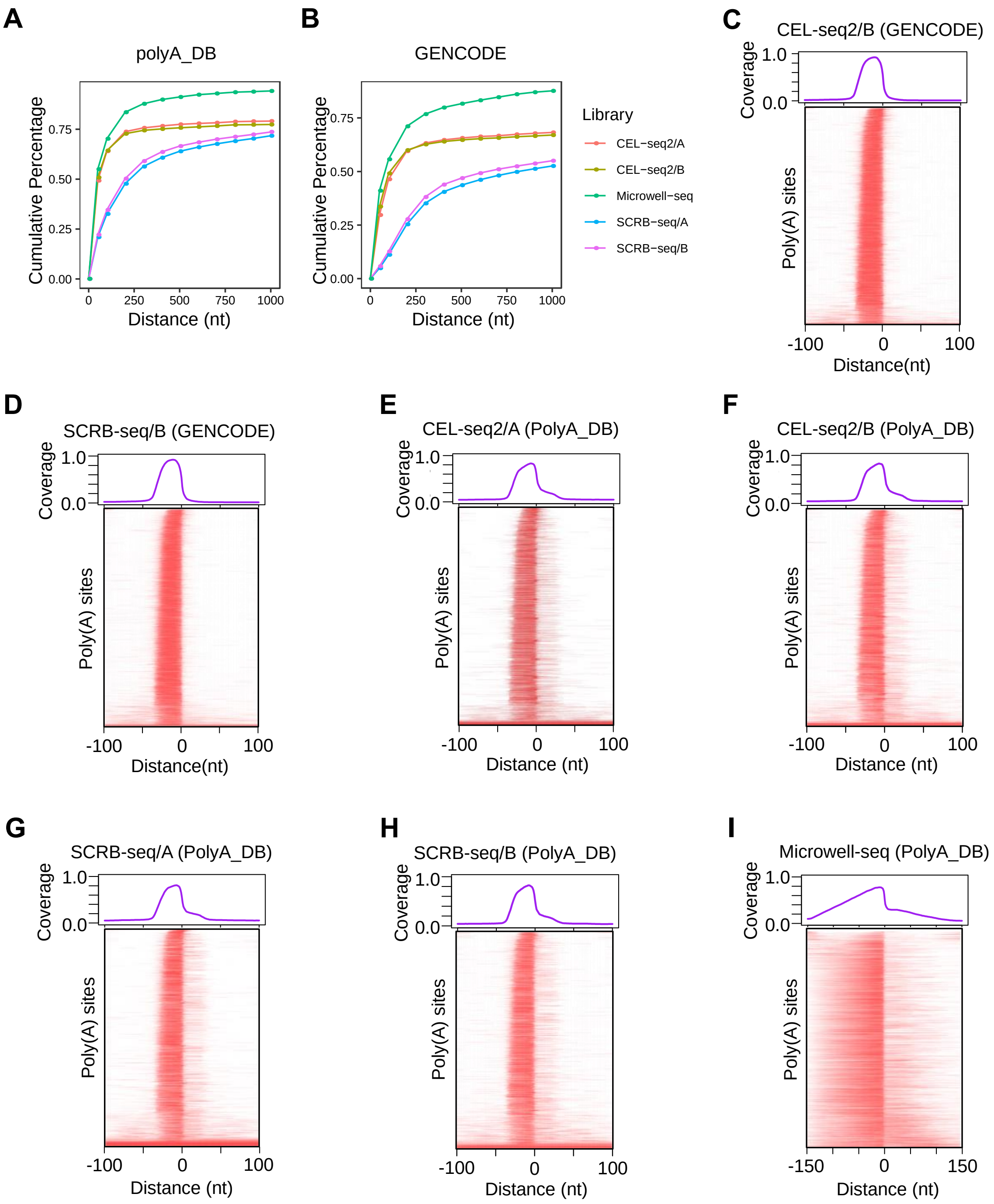

Figure S1. The distributions of poly(A) reads around poly(A) sites annotated in GENCODE and PolyA_DB_3 in 3'-tag based scRNA-seq data.

(A-B) The cumulative distributions of distances between 3' end of poly(A) reads and their closest annotated in PolyA_DB 3 **(A)** and GENCODE **(B)**. Y-axis represents cumulative percentage and

X-axis represents distance. Five 3'-tag based scRNA-seq datasets are used, including CEL-seq2/A, CEL-seq2/B. SCRB-seq/A, SCRB-seq/B and Microwell-seq.

(**C-D**) The plots as supplements to Fig. 1B show read coverage of poly(A) reads around poly(A) sites annotated in GENCODE in CEL-seq2/B **(C)** and SCRB-seq/B **(D)** dataset. The upper panels depict average read coverage of poly(A) reads around poly(A) sites. Y-axis represents the average read coverage and X-axis represents the distance from upstream 100 nt to downstream 100 nt to annotated poly(A) sites. The lower panels depict the read coverage for each poly(A) site using heatmaps.

(E-I) Read coverage of poly(A) reads around poly(A) sites annotated in PolyA_DB 3 in all five 3'-tag based scRNA-seq datasets are shown, including CEL-seq2/A **(E)**, CEL-seq2/B **(F)**, SCRB-seq/A **(G)**, SCRB-seq/B **(H)** and Microwell-seq **(I)** .

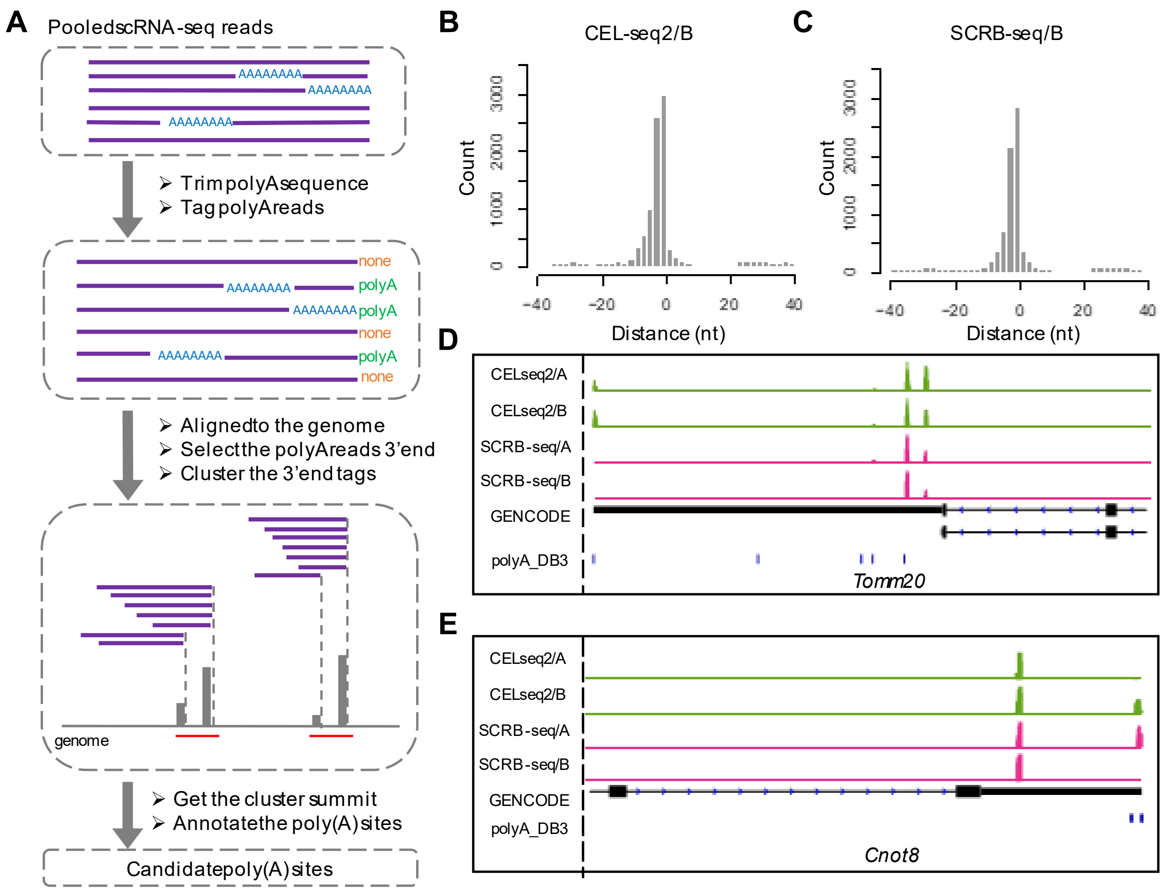

Figure S2. Identification of poly(A) sites using 3'-tag based scRNA-seq data.

(A) Schematic overview of SAPAS to identify poly(A) sites using 3'-tag based scRNA-seq data.

(B-C) The histograms as supplements to Fig. 1C-E depict comparisons between identified poly(A) sites and annotated poly(A) sites. The Y-axis represents the count of poly(A) sites and the X-axis represent the distance between identified poly(A) sites and the closest annotated poly(A) sites, **(B)** is for CEL-seq2/B dataset, **(C)** is for SCRB-seq/B dataset.

(D-E) The IGV plots depict examples of novel poly(A) sites identified in mESCs. Poly(A) reads coverages are displayed for *Tomm20* **(D)** and *Cnot8* **(E)**. Each row represents a 3'-tag based scRNA-seq dataset of mESCs.

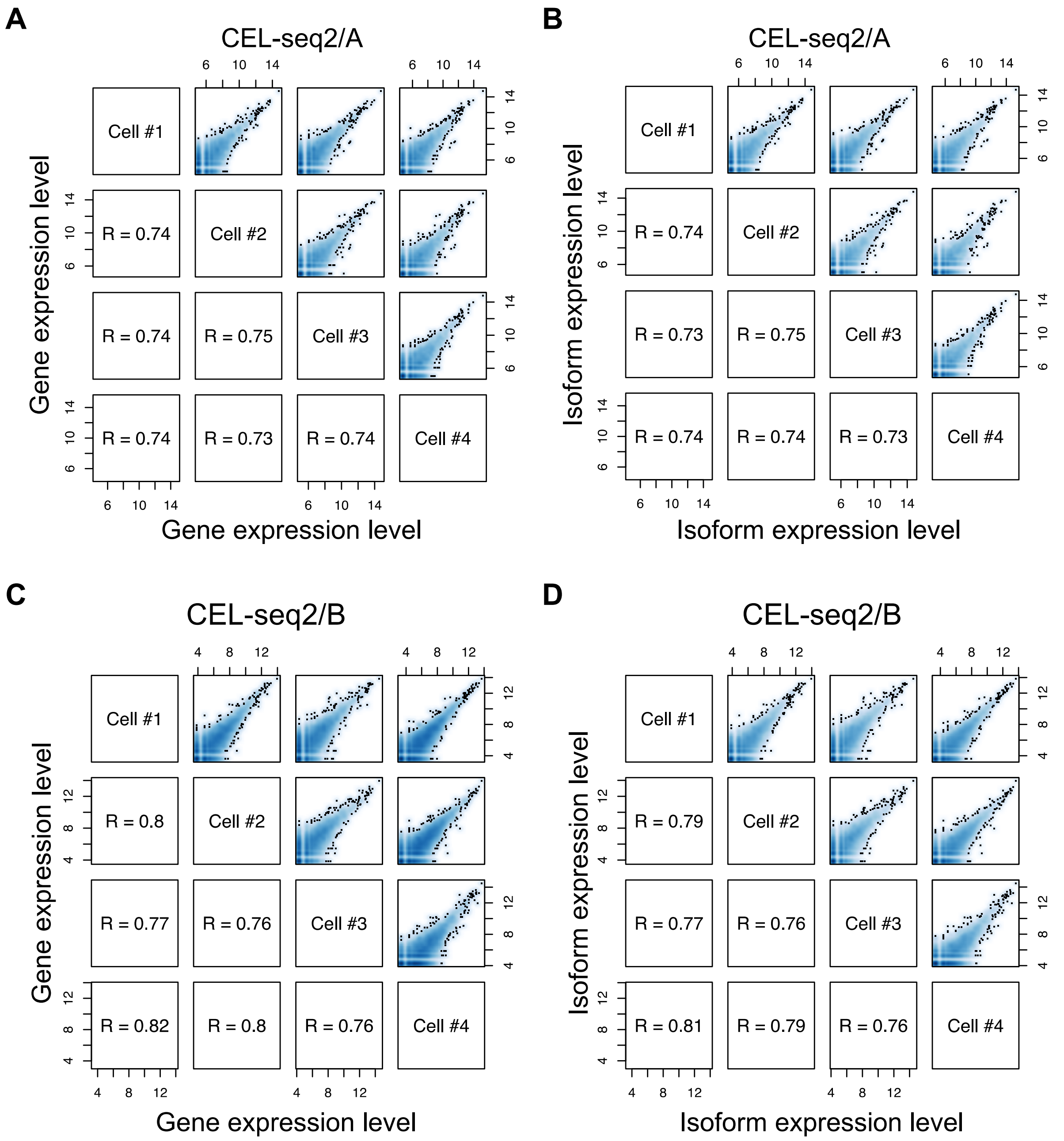

Figure S3. Pairwise comparisons of gene expression level and poly(A) isoform expression level for CEL-seq2.

The smooth scatterplots pairwise comparing gene expression level (**A, C**) and poly(A) isoform expression level (**B, D**) of 4 randomly selected single cells from CEL-seq2/A (**A, B**) and CEL-seq2/B (**C, D**) datasets. The lower left indicates the Pearson correlation coefficients (R) for each comparison.

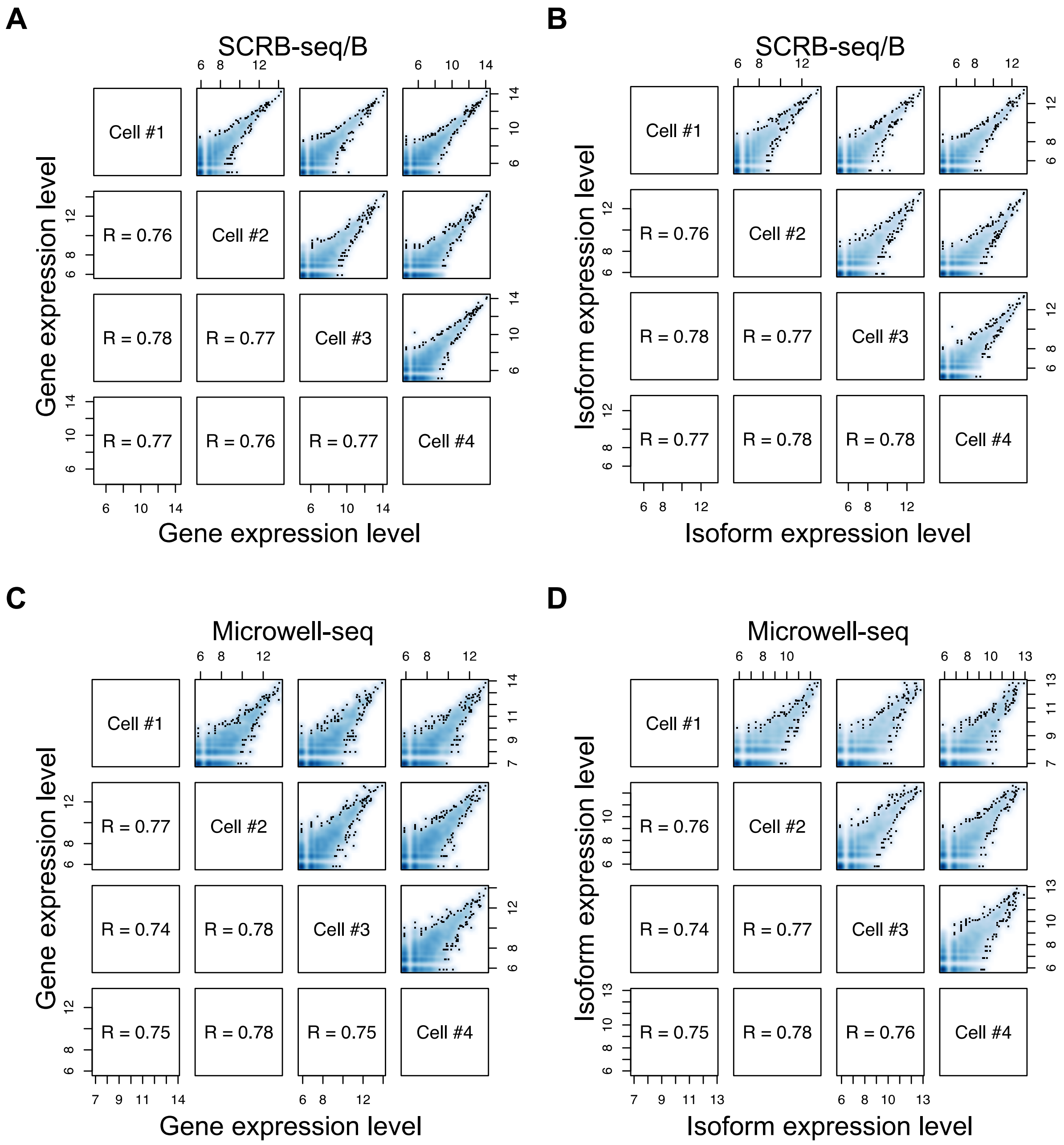

Figure S4. Pairwise comparisons of gene expression level and poly(A) isoform expression level for SCRB-seq and Microwell-seq.

The smooth scatterplots pairwise comparing gene expression level (**A, C**) and poly(A) isoform expression level (**B, D**) of 4 randomly selected single cells from SCRB-seq/B (**A, B**) and Microwell-seq (**C, D**) datasets. The lower left indicates the Pearson correlation coefficients (R) for each comparison.

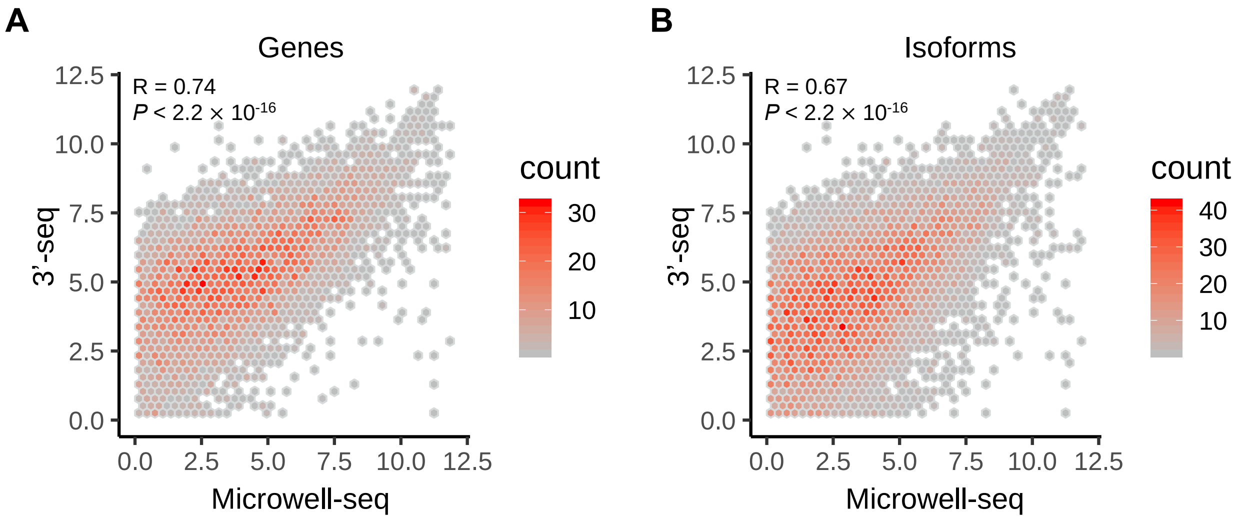

Figure S5. Comparisons of gene expression level and poly(A) isoform expression level estimated from Microwell-seq and 3'-seq data.

The hexbin scatterplots depict comparisons of gene expression level (**A**) and poly(A) isoform expression level (**B**) estimated from Microwell-seq and bulk 3'-seq. Y-axis represents bulk 3'-seq and X-axis represents Microwell-seq. The dashed lines represent the reference diagonal and the hexbins are colored by number of data points.

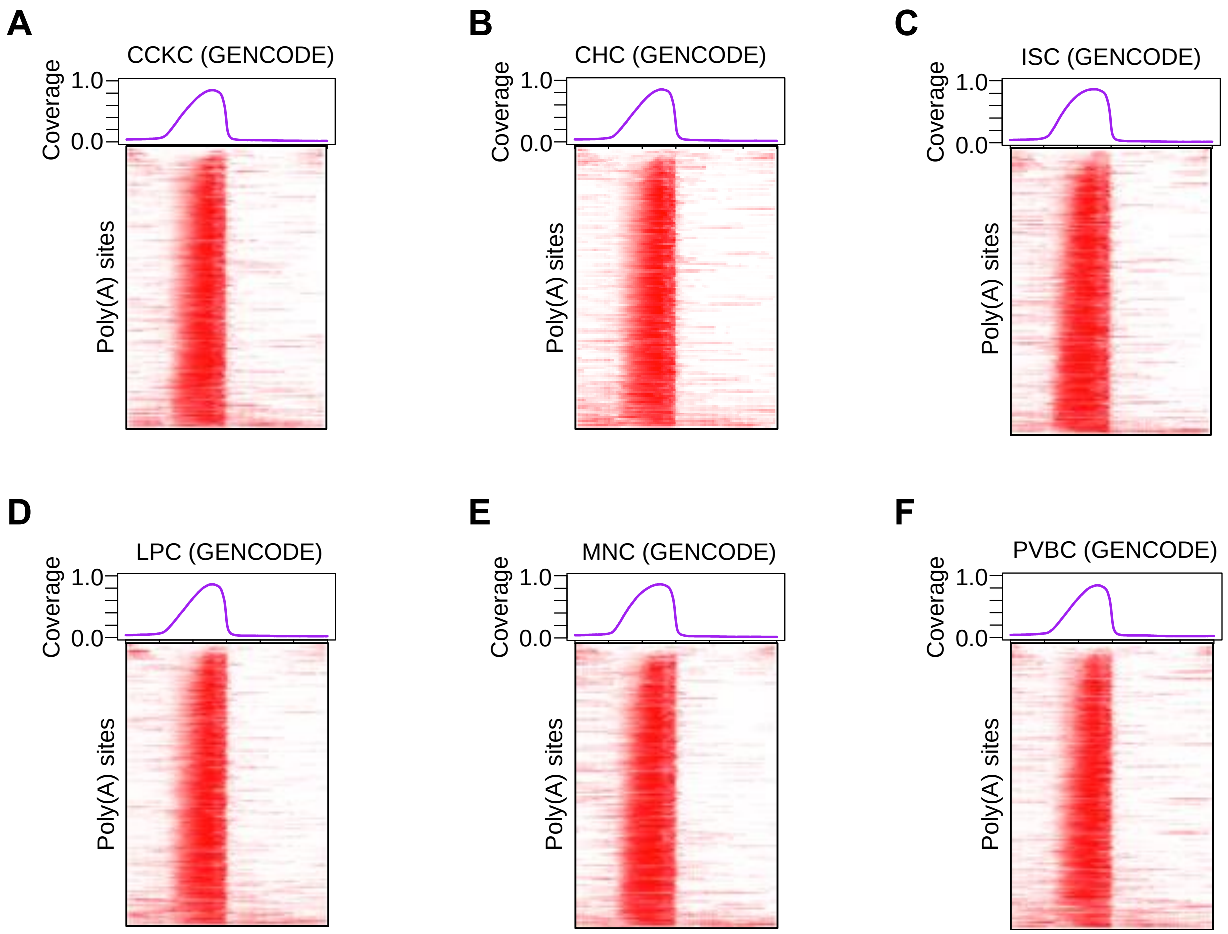

Figure S6. The distributions of poly(A) reads around poly(A) sites annotated in GENCODE in scRNA-seq data of GABAergic neurons.

Read coverage of poly(A) reads around poly(A) sites annotated in GENCODE in the scRNA-seq dataset of six different GABAergic neuron types are shown, including CCKC **(A)**, CHC **(B)**, ISC **(C)**, LPC **(D)**, MNC **(E)**, PVBC **(F)**. The upper panels depict average read coverage of poly(A) reads around poly(A) sites. Y-axis represents the average read coverage and X-axis represents the distance from upstream 100 nt to downstream 100 nt to annotated poly(A) sites. The lower panels showing the read coverage for each poly(A) site using heatmaps.

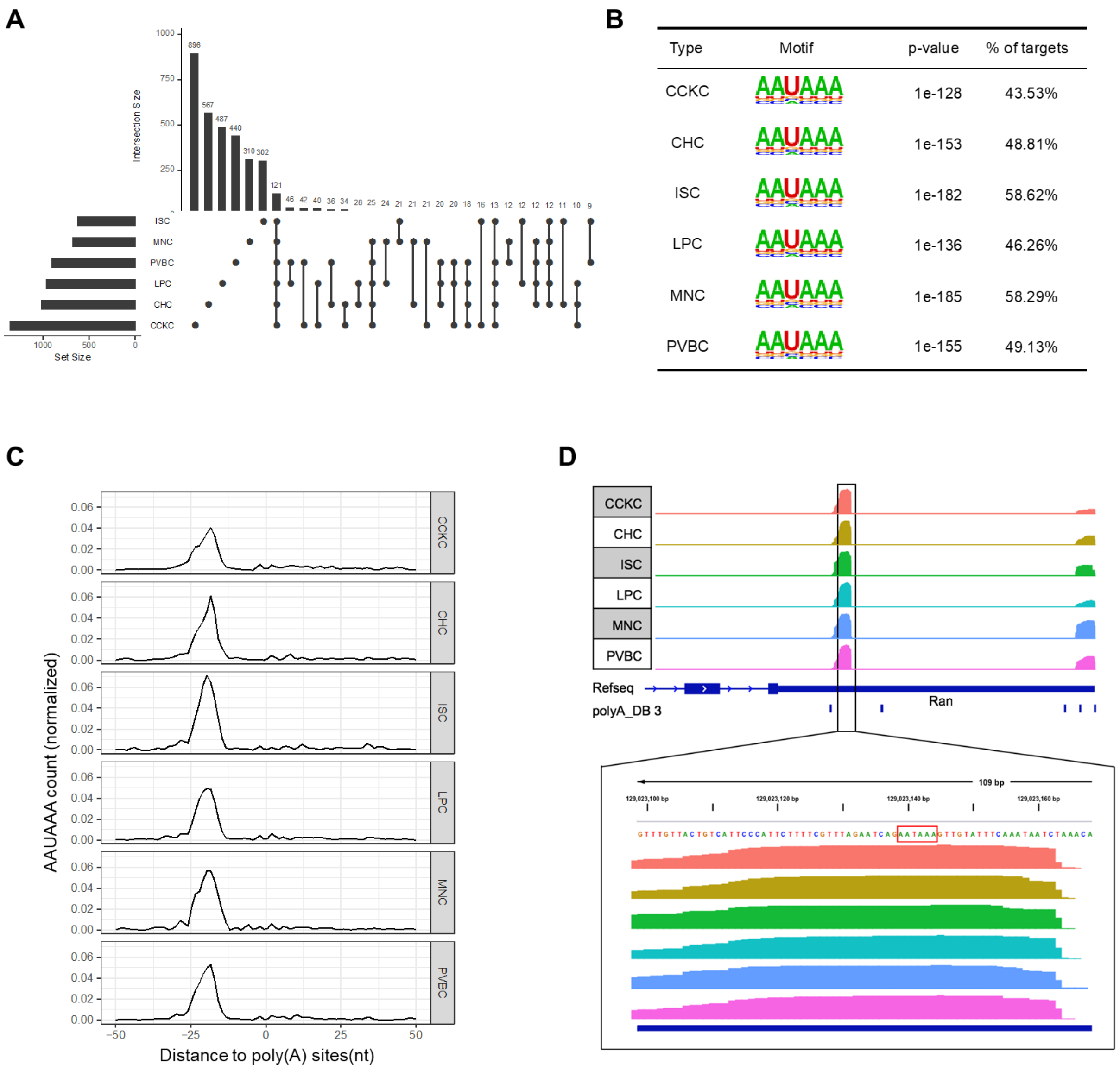

Figure S7. Novel poly(A) sites in GABAergic neurons.

(A) The Upset plot depicts the intersections of novel poly(A) sites identified in scRNA-seq data of different GABAergic neuron types (CCKC, CHC, ISC, LPC, MNC, PVBC).

(B) Canonical poly(A) signal (AAUAAA) enrichments for novel poly(A) sites identified in different GABAergic neuron types. *P*-values and percentages of targets are shown.

(C) The line plots illustrate the canonical poly(A) signal (AAUAAA) distribution from upstream 50 nt to downstream 50 nt to novel poly(A) sites. The Y-axis represents the frequency of the

canonical poly(A) signal (AAUAAA) and the X-axis represents the distance from upstream 50 nt to downstream 50 nt to novel poly(A) sites.

(D) A novel poly(A) identified in the 3' UTR of *Ran* in all six different GABAergic neuron types. The upper panel depicts read coverage of poly(A) reads along the 3' UTR of *Ran* and each row represents a specific GABAergic neuron type. Two annotation tracks are shown, including gene annotation model from Refseq and poly(A) site annotations from PolyA_DB 3. The lower panel shows that the novel poly(A) site harbors the canonical AAUAAA motif at ~20 nt upstream.

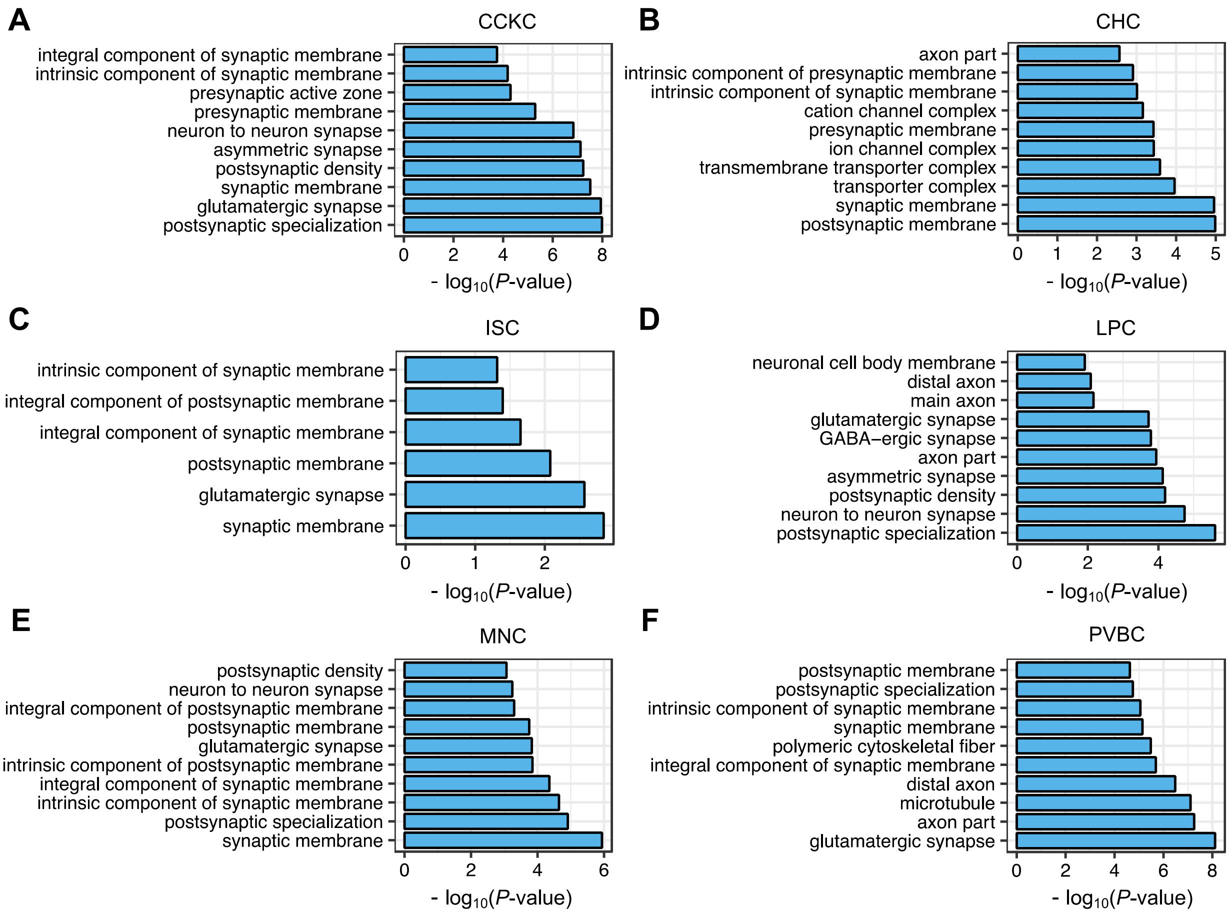

Figure S8. GO enrichment for genes with novel poly(A) sites.

The bar plots depict GO term (cellular component) enrichments for genes with novel poly(A) sites identified in 6 different GABAergic neuron types, including CCKC **(A)**, CHC **(B)**, ISC **(C)**, LPC **(D)**, MNC **(E)**, PVBC **(F)**. Y-axis represent GO term (cellular component) and X-axis represent the significance of GO term enrichment in $-\log_{10}$(P-value).

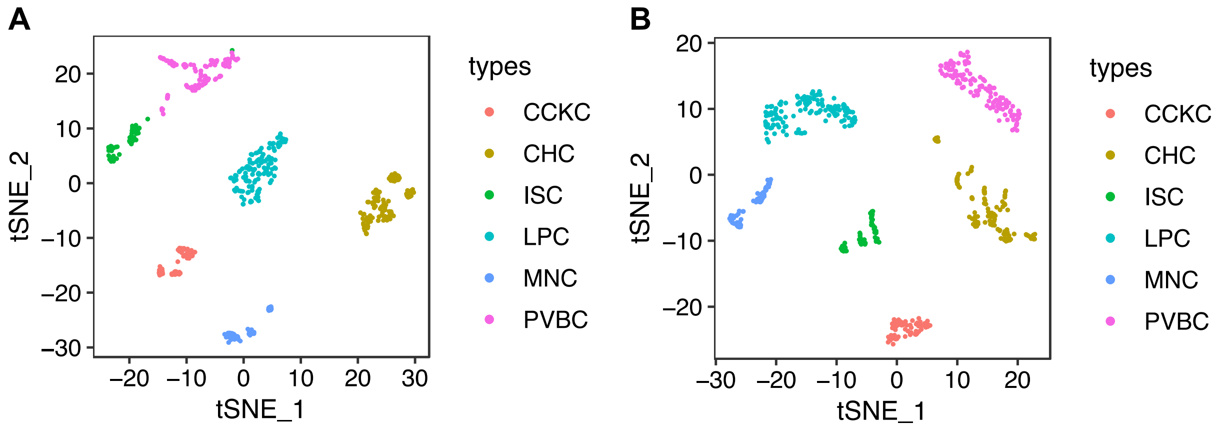

Figure S9. Clustering of GABAergic neurons.

As supplements to Fig. 3C, t-SNE plots of 6 different GABAergic neuron types on poly(A) isoform expression level **(A)** and poly(A) site usage **(B)**. The neuron types are labeled using different colors as indicated.

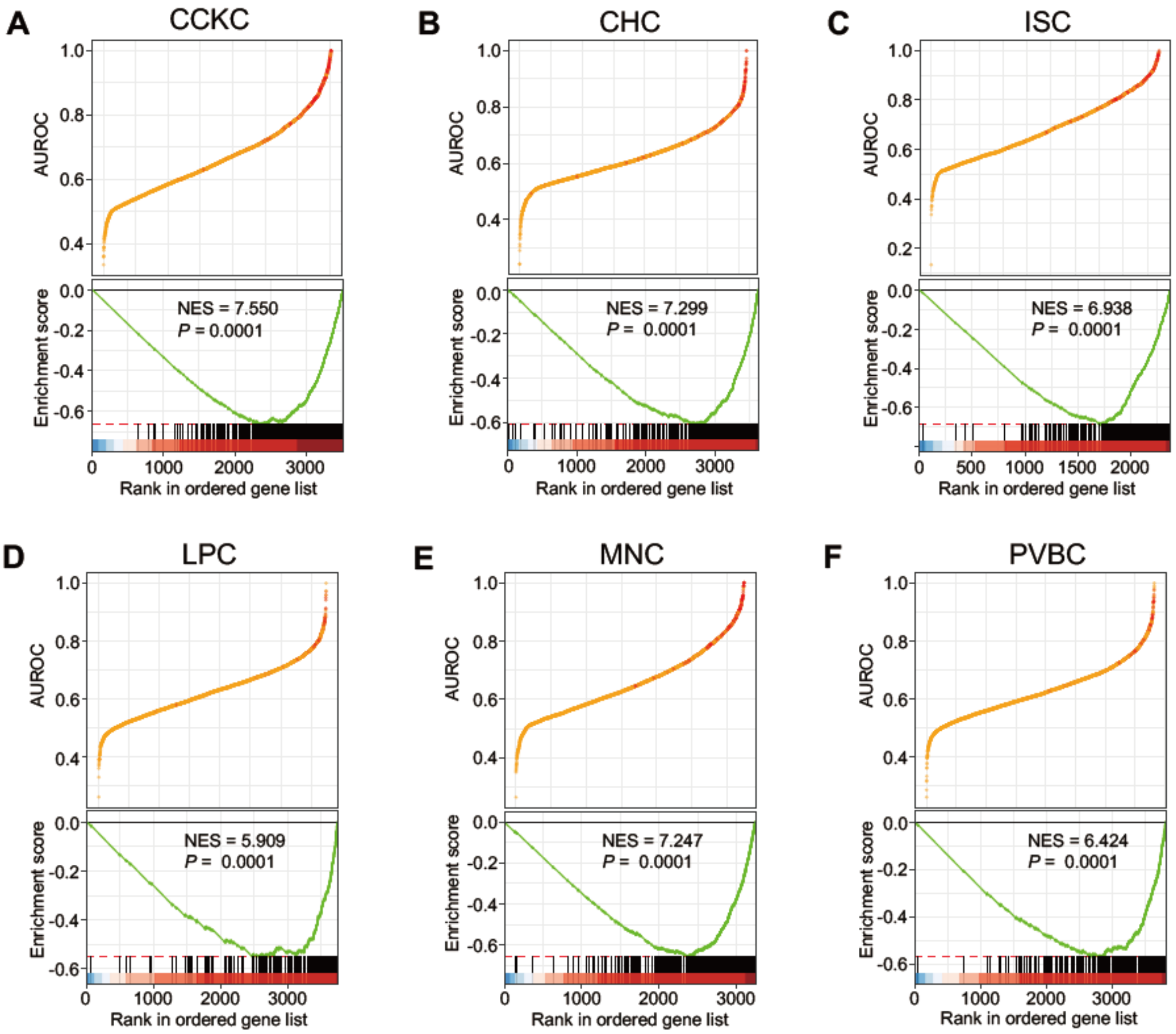

Figure S10. Comparisons of SAPAS and differential poly(A) usage analysis.

The plots depict that genes with significantly differential used poly(A) sites rank top on AUROC calculated by SAPAS in each GABAergic neuron type. The significantly differential used poly(A) sites were identified by differential usage analysis using one-versus-rest scheme for each GABAergic neuron type. The upper scatter plots show the ranks of genes with significantly differential used poly(A) sites. Y-axis represent AUROC and X-axis represent ranks of genes ordered by AUROC. Genes with significantly differential used poly(A) sites are labeled by red color. The lower panels show GSEA of genes with significantly differential used poly(A) sites. Genes are ranked by AUROC calculated by SAPAS in each GABAergic neuron type. The normalized enrichment scores (NES) and p-value are shown.

A

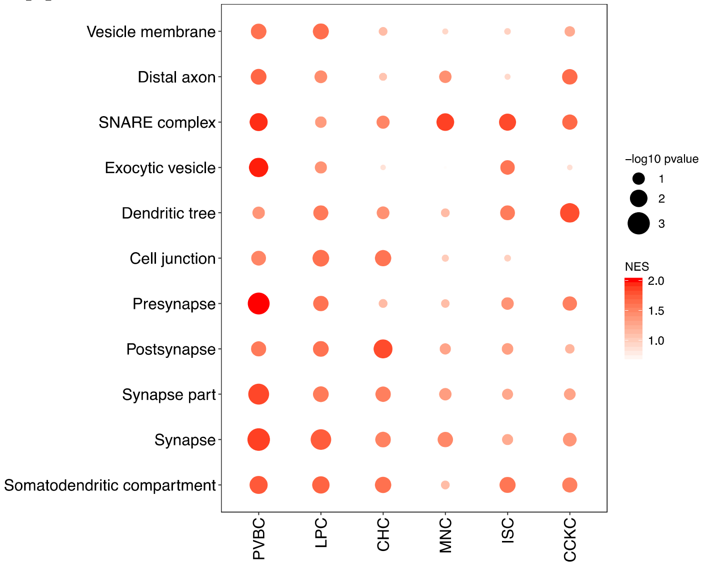

Figure S11. GO terms enriched in genes with cell-type specific APA events for each GABAergic neuron type.

As supplement to Fig. 3E, Synaptic communications related GO terms (cellular component) enriched in genes with cell-type specific APA events for each GABAergic neuron type. The normalized enrichment scores (NES) calculated by GSEA are indicated by gradient red and the p-values are indicated by circle size.

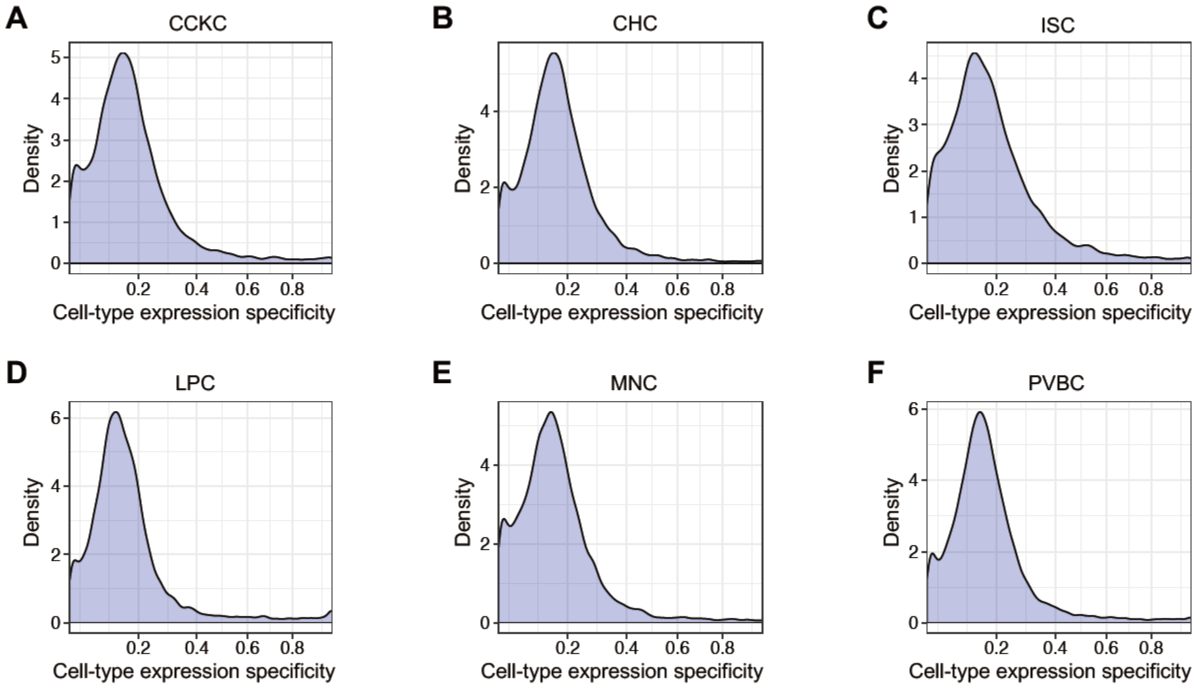

Figure S12. Cell-type expression specificity for each gene in different GABAergic neuron types.

The density plots depict cell-type expression specificity for each gene in different GABAergic neuron types, including CCKC **(A)**, CHC **(B)**, ISC **(C)**, LPC **(D)**, MNC **(E)**, PVBC **(F)**.

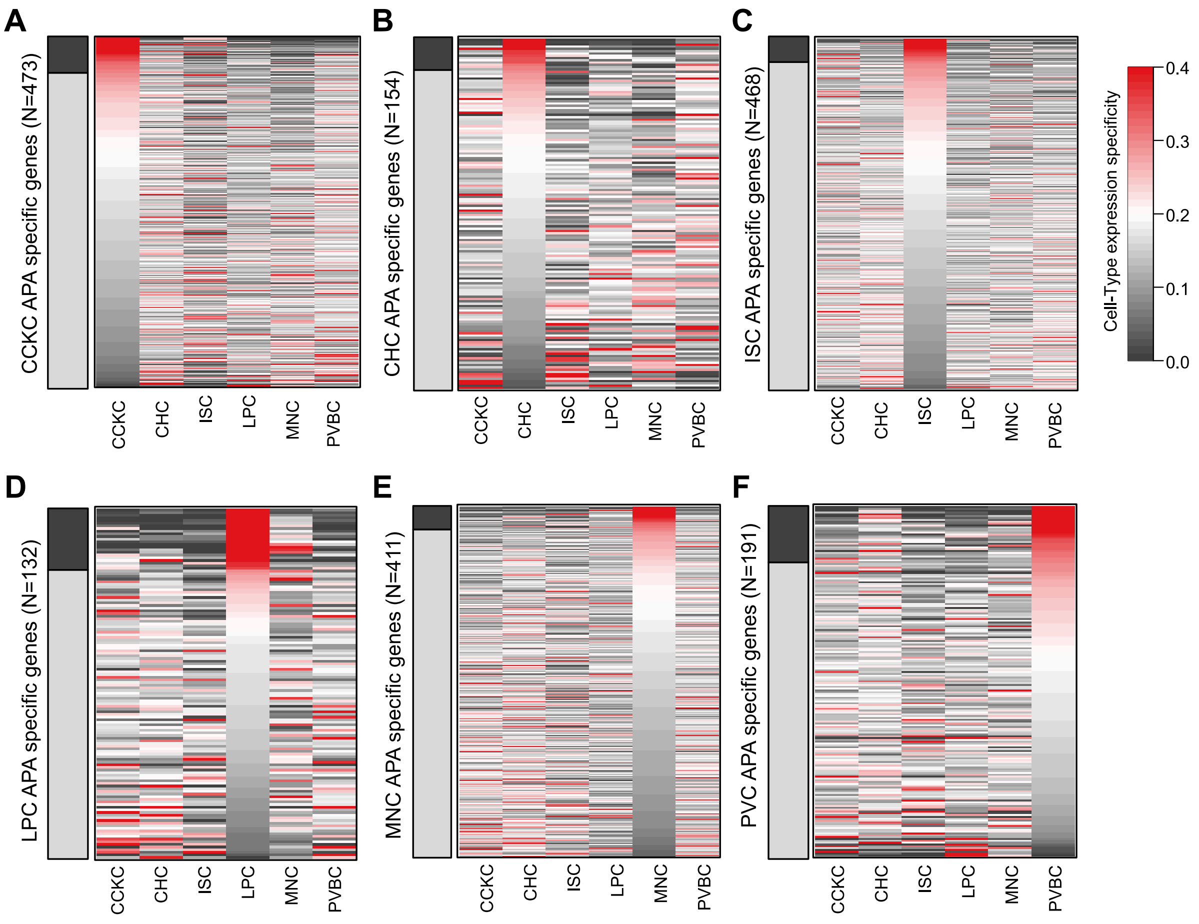

Figure S13. Cell-type expression specificity for genes with cell-type specific APA events. The heatmaps depict cell-type expression specificity for genes with cell-type specific APA events for each GABAergic neuron type. The cell-type expression specificity calculated by EWCE are shown in gradient grey to red. The left stacked bars show percentages of genes with cell-type expression specificity larger than 0.3 in black.

A

This study	Tasic 2018	#Single cells
CCKC	*Vip Crispld2 Htr2c*<br>*Vip Col15a1 Pde1a*	293
CHC	*Pvalb Vipr2*	89
ISC	*Vip Lmo1 Myl1*<br>*Vip Ptprt Pkp2*<br>*Vip Rspo1 Itga4*	619
LPC	*Sst Chodl*	179
MNC	*Sst Calb2 Pdlim5*	238
PVBC	*Pvalb Sema3e Kank4*	65

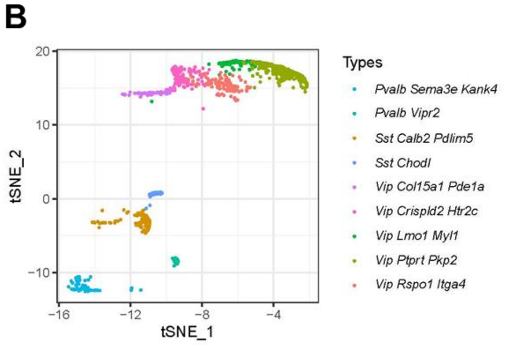

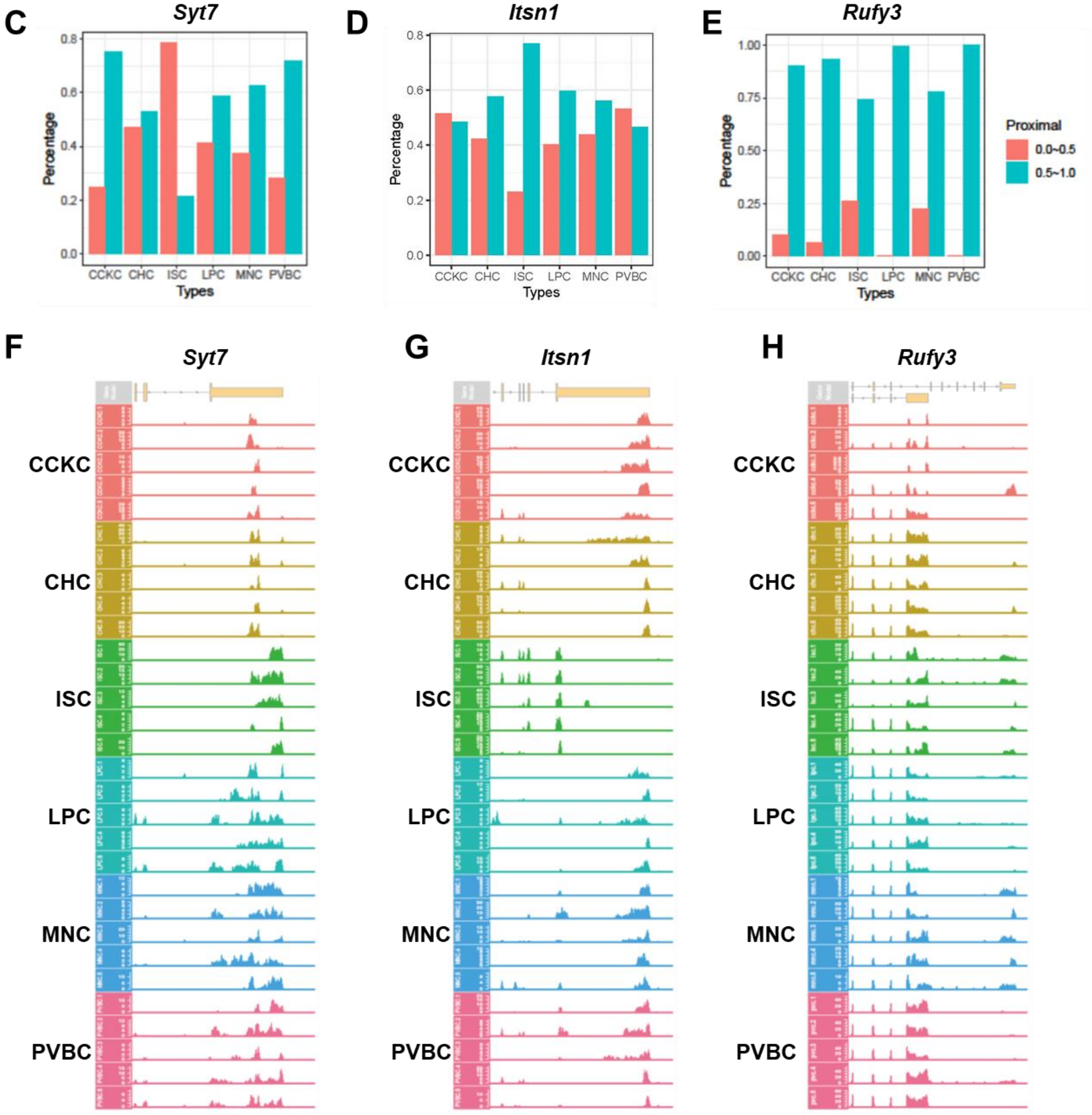

Figure S14. Validation of the cell-type specific APA events using a Smart-seq2 dataset from Tasic et al. study.

(A) Correspondence of neuron types in our study to Tasic et al. study. The number of single cells of each neuron type was shown.

(B) The t-SNE plot of all corresponded single cells based on the coordinated provided in Tasic et al. study. Different cell types are labeled using different colors as shown.

(C-E) The bar charts show the proportions of single cells with proximal poly(A) site usage at the range of 0~0.5 and 0.5~1 for the cell-type specific APA genes in Fig. 3, including *Syt7* **(C)**, *Itsn1* **(D)** and *Rufy3* **(E)**.

(F-H) The scRNA-seq read coverage of each cell-type specific APA gene from Smart-seq2 data of Tasic et al. study is displayed. Each track represents a single cell randomly selected for corresponded cell types. Different cell types are shown using different colors.

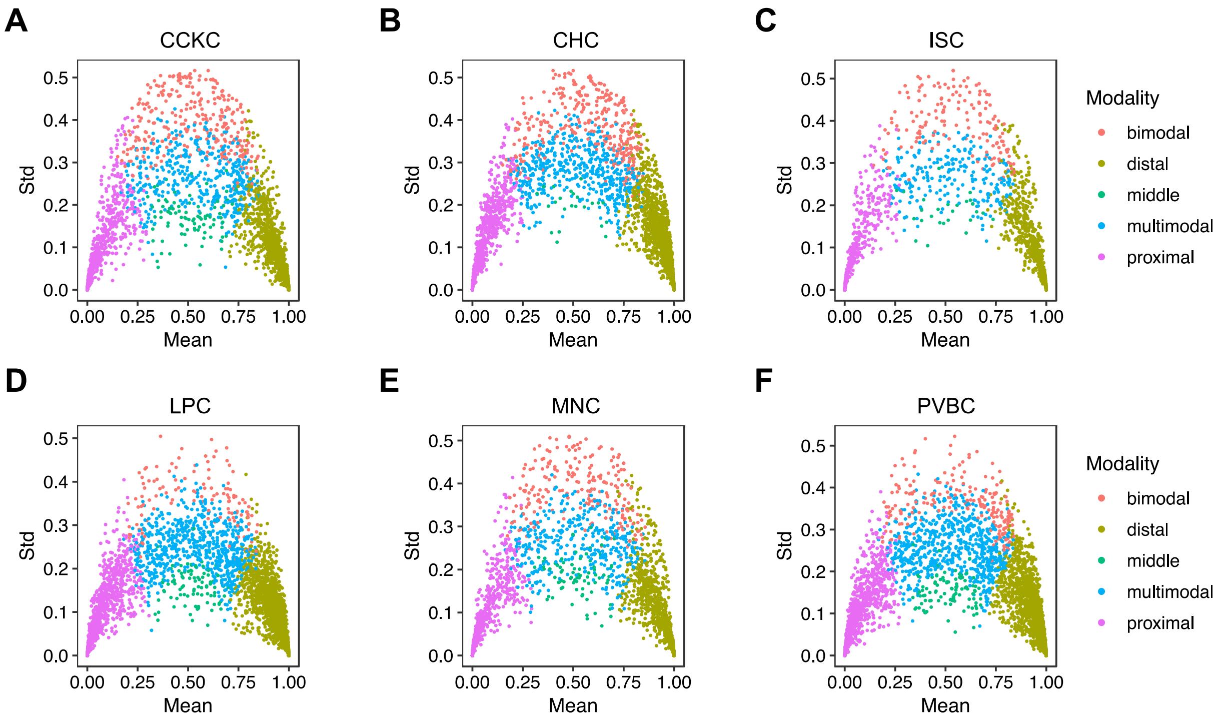

Figure S15. Mean-variance relations of distal poly(A) site usage in different GABAergic neuron types.

The scatter plots depict the standard deviation of distal poly(A) site usage across single cells as a function of the average of distal poly(A) site usage. 6 different GABAergic neuron types are shown, including CCKC **(A)**, CHC **(B)**, ISC **(C)**, LPC **(D)**, MNC **(E)**, PVBC **(F)**. The estimated modalities are labeled by different colors as indicated. Y-axis represents the standard deviation and X-axis represents mean.

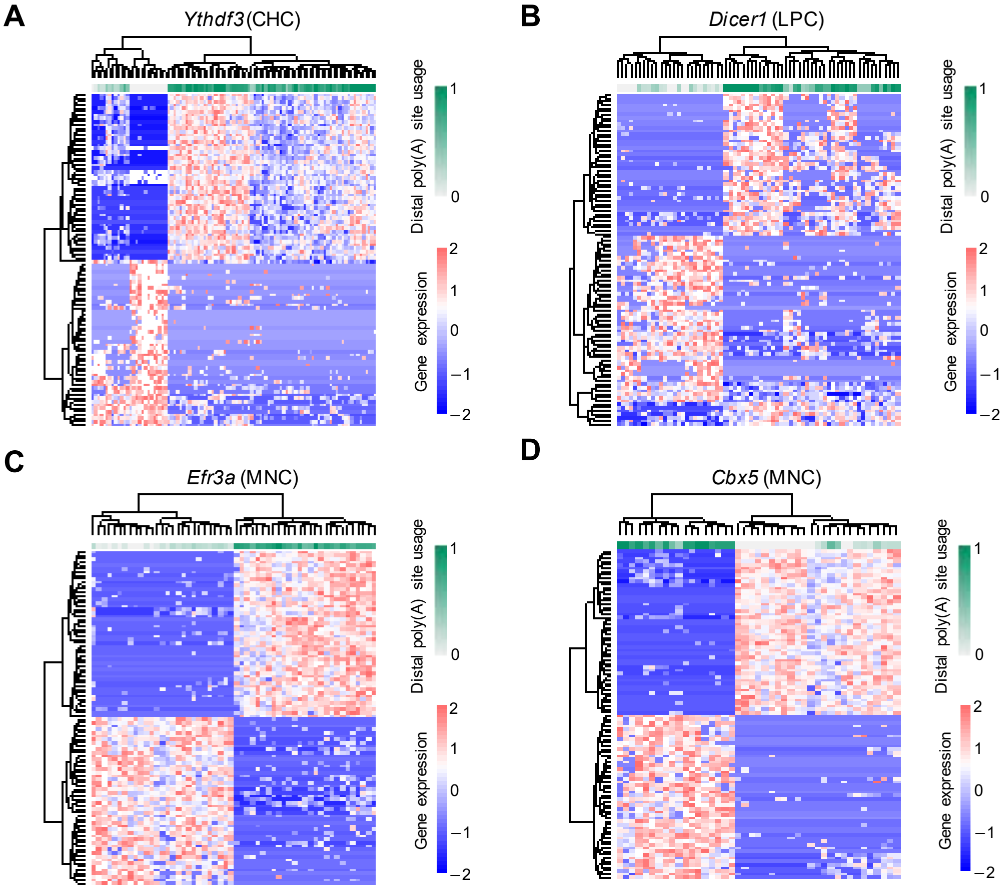

Figure S16. Examples with the potential to demarcate subpopulations of GABAergic neurons.

The heatmap depicts that those genes top correlated with distal poly(A) site usage could cluster specific GABAergic neuron type into subpopulations. **(A)** is for *Ythdf3* in CHC. **(B)** is for *Dicer1* in LPC. **(C)** is for *Efr3a* in MNC. **(D)** is for *Cbx5* in MNC. Columns represent single cells and rows represent correlated genes. The blue to red gradient represents gene expression level in $\log_2$(uTPM + 1). The green gradient represents distal poly(A) site usage of the gene.
